# Adipocyte β-arrestin-2 is essential for maintaining whole body glucose and energy homeostasis

**DOI:** 10.1038/s41467-019-11003-4

**Published:** 2019-07-03

**Authors:** Sai P. Pydi, Shanu Jain, Wesley Tung, Yinghong Cui, Lu Zhu, Wataru Sakamoto, Shalini Jain, Brent S. Abel, Monica C. Skarulis, Jie Liu, Thanh Huynh, Karel Pacak, Marc G. Caron, Oksana Gavrilova, Toren Finkel, Jürgen Wess

**Affiliations:** 1 Molecular Signaling Section, Laboratory of Bioorganic Chemistry, National Institute of Diabetes and Digestive and Kidney Diseases, Bethesda, MD NIH-20892 USA; 2 Mouse Metabolism Core, National Institute of Diabetes and Digestive and Kidney Diseases, Bethesda, MD 20892 USA; 3 Diabetes, Endocrinology, and Obesity Branch, National Institute of Diabetes and Digestive and Kidney Diseases, Bethesda, MD 20892 USA; 4 Center for Molecular Medicine, National Heart, Lung, and Blood Institute, Bethesda, MD 20892 USA; 5 Section on Medical Neuroendocrinology, Eunice Kennedy Shriver National Institute of Child Health and Human Development, Bethesda, MD 20892 USA; 6 Department of Cell Biology, Duke University Medical Center, Durham, NC 27710 USA

**Keywords:** Physiology, Metabolism, Fat metabolism, Diseases, Endocrine system and metabolic diseases

## Abstract

β-Arrestins are major regulators of G protein-coupled receptor-mediated signaling processes. Their potential roles in regulating adipocyte function in vivo remain unexplored. Here we report the novel finding that mice lacking β-arrestin-2 (barr2) selectively in adipocytes show significantly reduced adiposity and striking metabolic improvements when consuming excess calories. We demonstrate that these beneficial metabolic effects are due to enhanced signaling through adipocyte β3-adrenergic receptors (β3-ARs), indicating that barr2 represents a potent negative regulator of adipocyte β3-AR activity in vivo. Interestingly, essentially all beneficial metabolic effects caused by adipocyte barr2 deficiency are absent in adipocyte barr2-PRDM16 double KO mice, indicating that the metabolic improvements caused by the lack of barr2 in adipocytes are mediated by the browning/beiging of white adipose tissue. Our data support the novel concept that ‘G protein-biased’ β3-AR agonists that do not promote β3-AR/barr2 interactions may prove useful for the treatment of obesity and related metabolic disorders.

## Abstract (web summary)

Beta3-adrenergic receptor signaling regulates adipose tissue browning. Here, the authors show that barr2 regulates internalization of beta3-adrenergic receptors and that mice lacking barr2 in adipocytes are protected from diet-induced weight gain and metabolic complications.

## Introduction

Obesity represents a major risk factor for developing type 2 diabetes (T2D), cardiovascular disorders, immune dysfunction, various types of cancer, and several other severe human diseases^1^. The finding that adult humans harbor both classical brown adipose tissue (BAT) and brown-like or beige adipocytes in white adipose tissue (WAT) has generated immense interest in the obesity/metabolism field^2–8^. Brown/beige adipocytes utilize lipids and glucose as a fuel source and expend energy as heat, a process referred to as adaptive thermogenesis^4,7^. Importantly, the browning/beiging of WAT can counteract fat accumulation and improve whole body glucose homeostasis in experimental animals^9–12^. Taken together, these observations have stimulated intense efforts to develop novel anti-obesity drugs that are able to promote the browning/beiging of WAT depots^11,13,14^.

Adaptive thermogenesis function is predominantly mediated by UCP1 present in the inner mitochondrial membrane^11,14,15^. However, recent studies also implicate a creatine-driven substrate cycle in stimulating adaptive thermogenesis in beige adipocytes^16,17^. Upon cold exposure or hormonal or chemical stimulation, beige adipocytes can emerge in WAT depots^11,13,14,18^. Thus, a better understanding of the cellular pathways that regulate the appearance of beige fat is likely to stimulate the development of novel therapies for the treatment of obesity and its associated metabolic disorders.

Adipocyte function, like that of virtually all other cell types, is regulated by the activity of cell surface G protein-coupled receptors (GPCRs)^19^. For example, human subcutaneous fat expresses transcripts for more than 150 non-odorant GPCRs^19^. Importantly, activation of adipocyte β-adrenergic^4,14^ and A_2a_ adenosine receptors^20^, which primarily stimulate G_s_-mediated signaling, promotes the beiging of WAT and non-shivering thermogenesis.

The activity of most GPCRs is regulated by β-arrestin-1 and- 2 (barr1 and barr2, respectively), two intracellular proteins that bind to activated GPCRs, thus terminating GPCR signaling and promoting receptor internalization^21^. However, β-arrestins can also act as signaling molecules in their own right^22,23^. Various lines of evidence indicate that β-arrestins play important roles in regulating many key metabolic functions^24,25^. However, the observed in vivo phenotypes are difficult to interpret since the two β-arrestins are expressed in most tissues and cell types.

In this study, we used adipocyte-specific mutant mouse models to examine the potential role of barr2 in regulating adipocyte function and whole body glucose homeostasis. Strikingly, mice selectively lacking barr2 in adipocytes are protected against high fat diet (HFD)-induced weight gain and the associated metabolic deficits.

Our new data showing that barr2 acts as a strong negative regulator of adipocyte β3-adrenergic receptor (β3-AR) signaling should prove useful to design novel strategies to stimulate the browning/beiging of WAT for therapeutic purposes. For example, novel β-AR agonists that can activate G proteins with high potency but are unable to interact with β-arrestins (so-called ‘G protein-biased’ agonists) are predicted to promote beiging of WAT with considerably greater efficacy than non-biased β-AR agonists.

## Results

### Generation of adipocyte-selective barr2 knockout mice

**Fig. 1:**
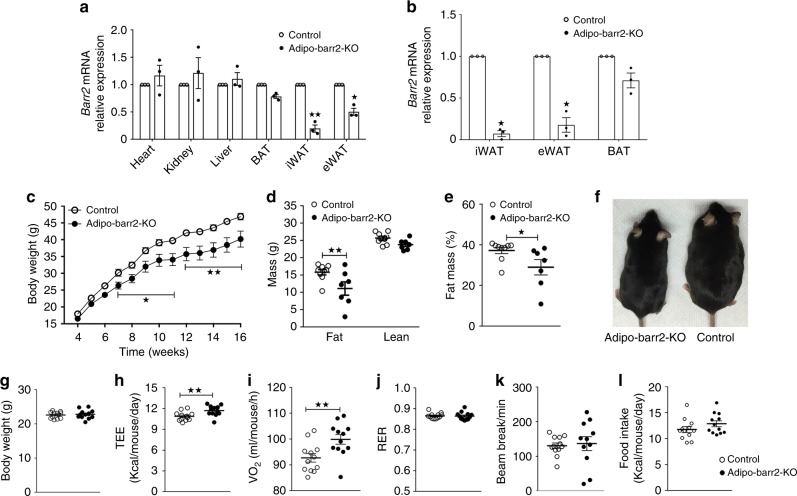
Mice lacking barr2 in adipocytes show decreased body weight and fat mass, and increased energy expenditure when maintained on a HFD. **a** Expression of *barr2* mRNA in different tissues of *adipo-Cre barr2 f/f* mice and control littermates (*barr2 f/f*). *Barr2* expression data are normalized relative to the expression of *β-actin* (*n* = 3 per group). For simplicity, we refer to the *adipo-Cre barr2 f/f* mice as adipo-barr2-KO mice throughout the manuscript. **b** Relative mRNA expression levels of *barr2* in isolated mature adipocytes from iWAT, eWAT, and BAT (*n* = 3 or 4 per group). **c** Body weights of control and adipo-barr2-KO mice consuming a HFD (*n* = 7–9 per group). **d**, **e** Body composition (**d**) and fat mass (% of body weight) (**e**) of mice maintained on a HFD for 12 weeks (*n* = 7–9 per group). **f** Images of representative control and adipo-barr2-KO KO mice (12-week-old males on HFD). **g**–**k** Metabolic studies during the initial phase of HFD feeding (first week; mouse age: 6–7 weeks). **g** Body weight. **h** Total energy expenditure (TEE). **i** Oxygen consumption rate (VO_2_). **j** Respiratory exchange ratio (RER). **k** Ambulatory activity. **l** Food intake. Measurements were carried out using the Oxymax/CLAMS system (*n* = 12 male mice per group). All data are expressed as means ± s.e.m. *p < 0.05, **p < 0.01 (**a**, **b**, **d**, **e**, **g**–**l** two-tailed Student’s *t*-test; **c** two-way ANOVA followed by Bonferroni’s post hoc test)

To examine the physiological function of barr2 in adipose tissue, we inactivated *barr2* selectively in adipocytes. Specifically, we crossed floxed barr2 mice (*barr2 f/f* mice)^26^ with *adipoq-Cre* mice, which express Cre recombinase under the control of the adipocyte-specific *adiponectin* promoter^27^. Figure 1a shows that *barr2* mRNA expression levels were greatly reduced in epididymal fat (eWAT) and subcutaneous inguinal fat (iWAT) of *barr2 f/f* mice carrying the *adipoq-Cre* transgene. In contrast, *barr2* transcript levels were only slightly reduced in BAT (Fig. 1a), in agreement with previous observations that the use of *adipoq-Cre* mice^27^ often results in a more efficient deletion of floxed genes in WAT, as compared to BAT (see, for example ref. ^16^). The expression of *barr2* in other metabolically relevant tissues was not altered in *adipoq-Cre barr2 f/f* mice, as compared to *barr2 f/f* control animals (Fig. 1a).

We also studied *barr2* expression levels using RNA prepared from purified primary mature adipocytes from three different fat pads (Fig. 1b). Adipocytes isolated from iWAT or eWAT from *adipoq-Cre barr2 f/f* mice showed a pronounced reduction (>80%) in *barr2* expression (Fig. 1b). In contrast, adipocytes from BAT displayed only a ~40% decrease in *barr2* transcript levels (Fig. 1b). The disruption of the *barr2* gene in adipocytes had no significant effect on *barr1* expression levels in different fat depots or other metabolically relevant tissues (Supplementary Fig. 1a). Consistent with the mRNA expression data, Western blotting studies indicated that barr2 protein was no longer detectible in iWAT but was still highly expressed in BAT of *adipoq-Cre barr2 f/f* mice (Supplementary Fig. 2). For simplicity, we refer to the *adipoq-Cre barr2 f/f* mice as ‘adipo-barr2-KO mice’ throughout this manuscript.

### Adipo-barr2-KO mice show reduced adiposity and enhanced energy expenditure

When control and adipo-barr2-KO mice were raised on standard chow, no significant differences in body weight and body composition were observed between the two mouse lines (Supplementary Fig. 1b-d).

To investigate a potential role of adipocyte barr2 in the development of obesity, control and adipo-barr2-KO mice (males) were maintained on a HFD at room temperature (23 °C) for several weeks (HFD feeding was initiated when the mice were 4 weeks old). Under these conditions, adipo-barr2-KO mice gained considerably less weight than their control littermates (Fig. 1c). After 16 weeks of HFD feeding, the mutant mice weighed ~15% less than the corresponding control mice (Fig. 1c, f). This decrease in body weight was associated with a significant reduction in fat mass in the barr2 mutant mice, as determined by magnetic resonance scanning (Fig. 1d, e). Lean body mass was not significantly different between the two groups (Fig. 1d).

We also measured energy expenditure, O_2_ consumption rate, food intake, and ambulatory activity in control and adipo-barr2-KO mice after 8 weeks of HFD feeding (23 °C). Interestingly, adipo-barr2-KO mice showed significantly increased total energy expenditure (TEE) when TEE was normalized by body weight (Supplementary Fig. 3a). However, TEE was significantly reduced when TEE was expressed as TEE/mouse/day (Supplementary Fig. 3b). There were no significant differences in respiratory exchange ratio (RER; ratio of CO_2_ produced to O_2_ consumed), ambulatory activity, and food intake between the two groups (Supplementary Fig. 3c–f).

To test the hypothesis that increased TEE was the cause of the reduced fat mass displayed by the mutant mice under the HFD regime, we conducted indirect calorimetry measurements at the beginning of the HFD feeding period when the two groups of mice did not differ in body weight (Fig. 1g). Under these conditions, adipo-barr2-KO mice also showed significant increases in TEE (Fig. 1h) and O_2_ consumption (ml/mouse/h) (Fig. 1i). In contrast, RER, locomotor activity, and food intake remained similar in control and mutant mice (Fig. 1j–l). The increase in energy expenditure displayed by the adipo-barr2-KO mice consuming the HFD was not observed when mice were maintained on regular chow (Supplementary Fig. 4).

We also measured cumulative food intake of 8–9-week-old HFD mice over a period of 12 days (mice were housed individually and food intake was measured every 3 days). This analysis showed that cumulative food intake during this period was very similar in control and adipo-barr2-KO mice (control, 48.2 ± 1.8 g; KO, 49.7 ± 1.9 g; *n* = 12 mice per group).

These data strongly suggest that increased energy expenditure causes the reduction in obesity displayed by the HFD mutant mice.

### Adipo-barr2-KO mice are protected against obesity-induced metabolic deficits

We next subjected control and adipo-barr2-KO mice to a series of in vivo metabolic tests. When mice were maintained on standard chow, we observed no significant differences in glucose and insulin tolerance tests between the two groups (Supplementary Fig. 5a, b). Moreover, fed and fasting blood glucose, fed plasma insulin, and plasma leptin levels were not affected by the *barr2* mutation (Supplementary Fig. 5c-e). We only noted a small decrease in plasma insulin levels in fasted adipo-barr2-KO mice (Supplementary Fig. 5d).

**Fig. 2:**
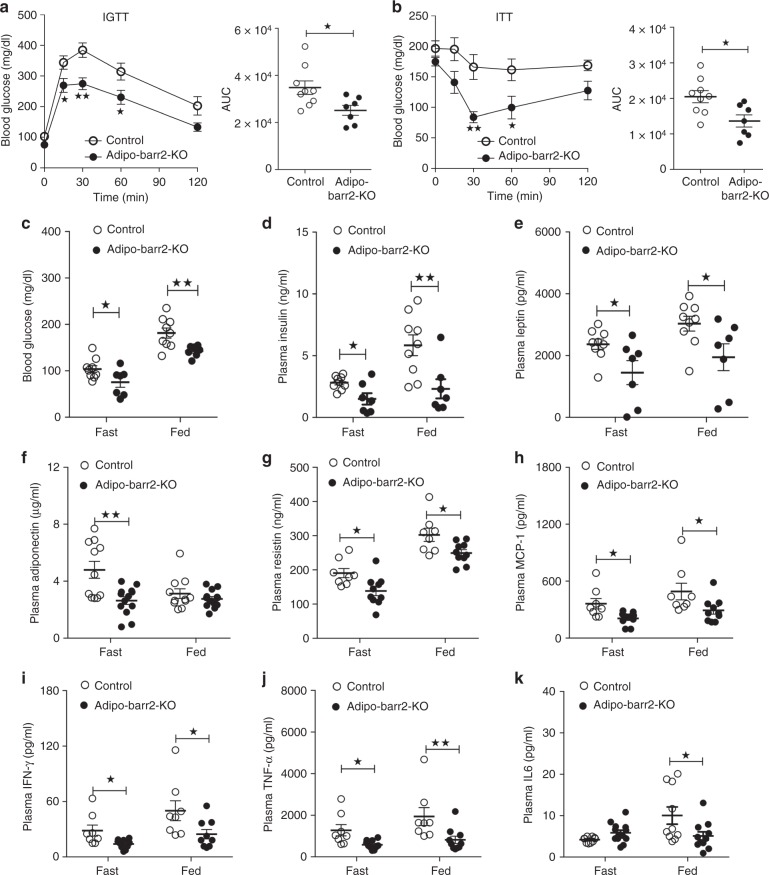
Adipo-barr2-KO mice are protected against the detrimental metabolic effects caused by the consumption of a HFD. **a**, **b** In vivo metabolic tests. Control and adipo-barr2-KO mice maintained on HFD for 8–9 weeks were subjected to i.p. glucose tolerance (1 g glucose/kg; IGTT) (**a**) and insulin tolerance tests (1 U insulin/kg i.p.; ITT) (**b**) (*n* = 7–9/group). AUC, area under the curve. **c**, **d** Freely fed and fasting blood glucose (**c**) and plasma insulin levels (**d**) of control and adipo-barr2-KO mice maintained on HFD for 8–9 weeks (*n* = 7–9/group). **e**–**g** Freely fed and fasting plasma leptin (**e**), adiponectin (**f**), and resistin (**g**) levels in control and adipo-barr2-KO mice maintained on HFD for 12 weeks (*n* = 7–10/group). **h**–**k** Plasma levels of proinflammatory cytokines. MCP-1 (**h**), IFN-γ (**i**), TNF-α (**j**), and IL6 (**k**) plasma levels in control and adipo-barr2-KO mice fed with HFD for 12 weeks (*n* = 8–11/group). All experiments were carried out with male mice. Data are given as means ± s.e.m. *p < 0.05, **p < 0.01 (**a**, **b** two-way ANOVA followed by Bonferroni’s post hoc test or two-tailed Student’s *t*-test (AUC); **c**–**k** two-tailed Student’s *t*-test)

A completely different pattern emerged when control and adipo-barr2-KO mice were maintained on a HFD for at least 8 weeks. Under these conditions, adipo-barr2-KO mice showed greatly improved glucose tolerance (Fig. 2a) and insulin sensitivity (Fig. 2b). Both fed and fasted barr2 mutant mice showed significantly reduced blood glucose levels, as compared to the corresponding control mice (Fig. 2c). At the same time, HFD adipo-barr2-KO mice displayed a pronounced reduction in plasma insulin levels under both fasting and fed conditions (Fig. 2d), consistent with the notion that the lack of WAT barr2 leads to enhanced insulin sensitivity. Taken together, these data indicate that adipo-barr2-KO mice are largely protected against the metabolic deficits associated with the consumption of a HFD.

We also measured plasma adipokine levels in HFD adipo-barr2-KO mice and control littermates. Plasma leptin and resistin levels were significantly reduced in HFD adipo-barr2-KO mice (Fig. 2e, g), consistent with the observation that these mice showed reduced adiposity (see Fig. 1d, e). Somewhat surprisingly, plasma adiponectin levels were also significantly decreased in the barr2 mutant mice (under fasting conditions only) (Fig. 2f). Moreover, the plasma levels of several proinflammatory cytokines, including MCP-1, IFN-γ, TNF-α, and IL6, were also significantly reduced in HFD adipo-barr2-KO mice (Fig. 2h–k).

### Adipo-barr2-KO mice show enhanced iWAT beiging

**Fig. 3:**
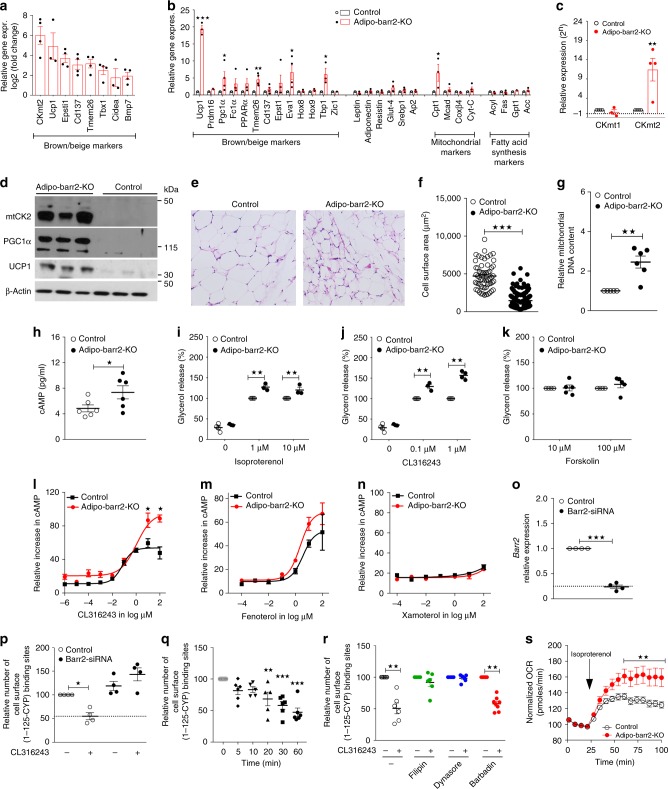
Lack of barr2 promotes WAT browning/beiging and enhances signaling through adipocyte β3-ARs. **a** Upregulation of genes promoting mouse iWAT browning/beiging in the absence of barr2 (RNA-seq analysis; HFD for 8 weeks; *n* = 3 or 4 per group). **b**, **c** Enhanced expression of genes involved in iWAT browning/beiging in the absence of barr2 (qRT-PCR analysis; HFD for 8 weeks; *n* = 3 or 4 per group). **d** Western blotting studies show striking increases in CKmt2, PGC-1α, and UCP1 protein levels in iWAT prepared from adipo-barr2-KO mice (HFD for 12 weeks). **e** Representative H&E-stained iWAT sections from iWAT (HFD for 16 weeks). **f** Reduction in adipocyte size in iWAT from adipo-barr2-KO mice (HFD for 16 weeks; *n* = 3 per group). **g** Mitochondrial DNA content (iWAT; HFD mice; *n* = 4 or 5 per group). **h** Tissue cAMP concentrations in iWAT (HFD mice; *n* = 6 per group). **i**–**k** Glycerol release studies. Mature adipocytes were stimulated with the indicated agents (*n* = 3 or 4 mice per group; diet: regular chow). **l**–**n** cAMP measurements. Mature adipocytes were incubated with β-AR subtype-selective agonists, followed by cAMP measurements: CL316243 (β3-selective; (**l**)), fenoterol (β2-selective; (**m**)), and xamoterol (β1-selective; (**n**)). Drug effects are expressed relative to cAMP responses caused by 100 μM forskolin (*n* = 4 or 5 mice per group; diet: regular chow). **o** Efficient reduction of *barr2* mRNA expression after treatment of 3T3-F442A cells with *barr2* siRNA (*n* = 4 per group). **p** Cell surface β3-AR expression levels after 30 min treatment of 3T3-F442A cells with CL316243 (1 μM). Prior to CL316243 incubation, cells had been treated with either *barr2* siRNA or scrambled control RNA (*n* = 4 per group). **q** Time course of disappearance of cell surface β3-ARs of 3T3-F442A cells stimulated with CL316243 (1 μM) (*n* = 3). **r** Incubation of 3T3-F442A cells with filipin (1 μg/ml) and dynasore (80 μM), but not barbadin (100 μM), prevents the loss of cell surface β3-ARs triggered by CL316243 (1 μM) treatment for 30 min (*n* = 3). **s** Oxygen consumption rate (OCR) of differentiated adipocytes derived from iWAT of adipo-barr2-KO mice is significantly increased after isoproterenol (10 μM) treatment. Normalized data are shown (basal levels = 100%) (*n* = 3). Male mice were used for all studies. Data are given as means ± s.e.m. *p < 0.05, **p < 0.01, ***p < 0.001 (**b**, **c**, **f**–**q** two-tailed Student’s *t*-test; **r** two-way ANOVA followed by Bonferroni’s post hoc test)

To identify cellular pathways that are altered by the lack of barr2 in iWAT, we prepared iWAT RNA from adipo-barr2-KO and control mice maintained on HFD for 8 weeks. The RNA samples were then subjected to RNA-seq analysis. Overall, ~2000 genes were upregulated and ~550 genes were downregulated in iWAT lacking barr2 (cutoff: log2 fold change ≥1.5 (Supplementary Fig. 6a, b). Most strikingly, this analysis showed that the expression of many key marker genes indicative of iWAT beiging, including *Ucp1*, *Cidea*, *Tmem26*, *CD137*, *Tbx1*, *Epsti1, Bmp7*, and *CKmt*2, was significantly increased in barr2-deficient iWAT (Fig. 3a). We also observed significant changes in the expression levels of various mitochondrial adipocyte marker genes and of genes involved in lipid metabolism (Supplementary Fig. 6b). qRT-PCR studies confirmed that barr2 deficiency in iWAT caused robust increases in the expression levels of genes critically involved in iWAT browning/beiging (Fig. 3b, c). In contrast to iWAT, BAT isolated from HFD control and adipo-barr2-KO mutant mice did not show any significant changes in thermogenic marker genes (Supplementary Fig. 6c). In eWAT lacking barr2, the expression of only some browning/beiging-associated genes was elevated, including *Tbx1* and *CKmt2* (Supplementary Fig. 6d).

In agreement with the gene expression data, the protein expression levels of Ucp1, CKmt2, and Pgc1α were dramatically increased in iWAT lysates prepared from HFD adipo-barr2-KO mutant mice (Fig. 3d). We also observed that creatine kinase activity was significantly enhanced in mitochondria from iWAT of adipo-barr2-KO mice, as compared to mitochondrial preparations from iWAT of control littermates (Supplementary Fig. 7).

H&E staining experiments showed that adipocyte size was greatly reduced in iWAT lacking barr2 (Fig. 3e, f). While the beiging of iWAT in young, lean mice consuming regular chow leads to the appearance of multilocular adipocytes, this morphological change is usually absent in iWAT undergoing beiging under a HFD regime (Patrick Seale, personal communication) (Fig. 3e).

To quantitate iWAT mitochondrial DNA content, we determined the ratio of mitochondrial to genomic DNA by real-time PCR (see Methods for details). As expected, this analysis revealed a significant increase in mitochondrial DNA levels in iWAT of HFD adipo-barr2-KO mice, as compared to HFD control mice (Fig. 3g).

In contrast to the findings obtained with iWAT from HFD adipo-barr2-KO mice, UCP1 and PGC1α protein levels were similar in BAT from HFD adipo-barr2-KO and control animals (Supplementary Fig. 8a). Moreover, total BAT weight did not differ significantly between the two groups of mice (Supplementary Fig. 8b). We also treated mature adipocytes from adipo-barr2-KO and control mice groups with CL316243, a selective β3-AR agonist known to enhance BAT activity by stimulating triglyceride breakdown in a cAMP-dependent fashion^28–30^. Biochemical experiments showed that CL316243 stimulated lipolysis and increased intracellular cAMP levels to a similar extent in mutant and control BAT cells (Supplementary Fig. 8c, d). These observations are consistent with the finding that barr2 expression remained high in BAT from adipo-barr2-KO (Fig. 1a, b), suggesting that classical BAT does not play a critical role in causing the metabolic phenotypes displayed by the HFD adipo-barr2-KO mice.

### Barr2 inhibits signaling through β3-ARs in mouse iWAT

WAT and BAT are richly innervated by sympathetic nerves, and increased activity of the sympathetic nervous system (SNS) triggers lipolysis in WAT and thermogenesis in BAT or beige fat^30^. These SNS functions are mediated by activation of β-ARs which belong to the superfamily of biogenic amine GPCRs^30^. The β-AR family comprises three distinct members, β1, β2, and β3. All three receptors are expressed by mouse adipocytes, although the β3-AR represents the predominant β-AR receptor subtype in murine adipocytes (β3 > β2 > β1)^30^. Importantly, activation of β-ARs induces browning/beiging of WAT, a response that is most easily detectable in iWAT^11,13,14,28^.

Since barr2 is known to bind to activated β-ARs^21^, we hypothesized that the lack of barr2 may lead to enhanced β-AR signaling in adipocytes of adipo-barr2-KO mice. To test this hypothesis, we first measured cAMP levels in iWAT from HFD adipo-barr2-KO and control mice (note that β-AR signaling stimulates adenylyl cyclase via activation of G_s_). We found that cAMP levels were significantly elevated in iWAT lacking barr2 (Fig. 3h). Increased β-AR signaling in adipocytes also promotes lipolysis via cAMP-dependent pathways^30^. To examine whether β-AR-mediated lipolysis was altered in the absence of barr2, we stimulated mature, isolated adipocytes from iWAT of adipo-barr2-KO and control mice with two different β-AR agonists, isoproterenol and CL316243. While isoproterenol is an agonist at all three β-AR subtypes, CL316243 selectively activates β3-ARs^29^. We found that both agonists stimulated lipolysis to a significantly greater extent in iWAT-derived adipocytes lacking barr2 than in control adipocytes (Fig. 3i, j).

To exclude the possibility that barr2 interfered with lipolytic processes by modulating intracellular pathways in a receptor-independent fashion, we stimulated mature, iWAT-derived adipocytes with forskolin, a direct activator of adenylyl cyclase. We found that forskolin (10 and 100 μM) stimulated lipolysis to a similar extent in control and barr2-deficient adipocytes (Fig. 3k), supporting the concept that barr2 acts upstream of adenylyl cyclase, most likely at the β-AR level.

To identify the β-AR subtype responsible for the observed increase in cAMP production in barr2-deficient adipocytes, we treated mature, iWAT-derived mutant and control adipocytes with subtype-selective β-AR agonists (β3, CL316243; β2, fenoterol; β1, xamoterol), followed by the measurement of intracellular cAMP levels. The β3-AR agonist CL316243 showed significantly greater efficacy in barr2-deficient adipocytes, as compared to control adipocytes (Fig. 3l). The β2-AR selective agonist fenoterol stimulated cAMP production in a similar fashion in control and KO adipocytes (Fig. 3m), while xamoterol (selective agonist for β1-ARs) was essentially devoid of functional activity in both groups of adipocytes (Fig. 3n).

Taken together, these data strongly suggest that barr2 deficiency selectively enhances signaling through β3-ARs in mouse adipocytes (iWAT).

### Barr2 mediates β3-AR internalization in adipocytes

Previous studies with cultured cells demonstrated that activated β3-ARs are unable to interact with β-arrestins^31,32^, providing a possible explanation for several reports that β3-ARs do not undergo activity-dependent internalization or desensitization^31,33,34^. For this reason, the β3-AR is generally considered an ‘atypical’ member of the GPCR superfamily^30,31,33,34^.

Given the fact that barr2 deletion in adipocytes greatly enhanced agonist-induced signaling through β3-ARs (Fig. 3l), we speculated that barr2 regulation of β3-AR internalization might occur in adipocytes, in contrast to other cell types studied previously. To address this question, we carried out receptor internalization studies using mouse 3T3-F442A adipocytes that express almost exclusively the β3-AR subtype^35^. After treatment of 3T3-F442A cells with either control siRNA or *barr2* siRNA (Fig. 3o), we stimulated cells with CL316243 (1 μM) at 37 °C for 30 min. Subsequently, we selectively labeled cell surface β3-ARs with [I^125^]-cyanopindolol ([I^125^]CYP, 1 nM) at 4 °C (please note that CYP acts as a membrane-impermeable ligand at 4 °C;^36^ see Methods for details). In control cells, CL316243 stimulation led to a pronounced loss (internalization) of ~50% of cell surface β3-ARs (Fig. 3p). In striking contrast, CL316243-induced β3-AR internalization was completely abolished in 3T3-F442A cells following *barr2* knockdown (Fig. 3p). A time course of the disappearance of cell surface β3-ARs of CL316243 (1 μM)-treated 3T3-F442A cells is shown in Fig. 3q. Interestingly, CL316243-induced β3-AR internalization remained unaffected by treatment of 3T3-F442A cells with barbadin (100 μM), a small molecule that selectively inhibits β-arrestin-mediated GPCR endocytosis via clathrin-coated pits^37^ (Fig. 3r). On the other hand, β3-AR sequestration was completely prevented by incubation of cells with filipin (1 μg/ml) or dynasore (80 μM) (Fig. 3r). Dynasore is an inhibitor of dynamin that interferes with both clathrin-dependent and -independent endocytic pathways^38^. In contrast, filipin is a cholesterol-binding agent that is able to prevent caveolae/lipid raft-mediated GPCR endocytosis, a process that is also dynamin-dependent^39^. Taken together, our findings suggest that the activated β3-ARs are internalized via a caveolae/lipid raft-dependent pathway in fat cells.

Using the same experimental strategy, we also studied agonist-induced β3-AR internalization in HEK 293 T transiently expressing the mouse β3-AR. In contrast to our findings with 3T3-F442A mouse adipocytes, treatment of β3-AR-expressing HEK 293 T cells with CL316243 (1 μM; 37 °C for 30 min) failed to induce β3-AR internalization (Supplementary Fig. 9a). For control purposes, we demonstrated that isoproterenol (10 μM) stimulation of HEK 293 T cells transiently expressing the human β2-AR resulted in robust β2-AR internalization (Supplementary Fig. 9b). These data support the novel concept that barr2 regulation of β3-AR internalization is indeed cell-type-specific.

We also studied whether the absence of barr2 affected β3-AR expression levels in native mouse fat tissue. Specifically, we used conditions that allowed us to selectively label β3-ARs in plasma membranes prepared from iWAT of adipo-barr2-KO and control mice (radioligand: [I^125^]CYP, 1 nM). Although there was a trend towards increased β3-AR expression levels in barr2-deficient preparations, this difference failed to reach statistical significance (*p* = 0.37) (control: 22 ± 4 fmol/mg, *n* = 11; adipo-barr2-KO: 32 ± 10, *n* = 9; means ± s.e.m.).

### Norepinephrine release is unchanged in adipo-barr2-KO mice

We next wanted to exclude the possibility that sympathetic tone is enhanced in adipo-barr2-KO mice, thus triggering enhanced β3-AR signaling in adipocytes. To address this issue, we measured norepinephrine (NE) levels in iWAT and urine from adipo-barr2-KO mice and control littermates. We found that iWAT and 24 h urine NE levels did not differ significantly between the two groups (Supplementary Fig. 10).

We also measured noradrenergic turnover in iWAT of HFD adipo-barr2-KO mice and control littermates by using a previously published method^40^. Specifically, the sums of the concentrations of NE, dopamine, DOPA (3,4-dihydroxyphenylalanine, an NE precursor), and DHPG (3,4-dihydroxyphenylglycol, an NE metabolite) were calculated and used as an index of noradrenergic turnover^40^. This analysis showed that the NE turnover index was very similar in iWAT of HFD adipo-barr2-KO and control mice (KO, 48.4 ± 3.2 ng/μg tissue; control, 48.0 ± 5.7 ng/μg tissue; six male mice per group). These results indicate that the lack of barr2 had no significant effect on NE release/turnover in iWAT of HFD adipo-barr2-KO mice.

### β-AR activation promotes O_2_ consumption in barr2-deficient adipocytes

In adipocytes, increased adrenergic signaling is known to increase mitochondrial O_2_ consumption^41,42^. Consistent with the data shown in Fig. 3h–j, isoproterenol (10 μM at 37 °C) and CL316243 (1 μM at 37 °C) treatment of differentiated adipocytes derived from iWAT of adipo-barr2-KO mice caused a significant increase in mitochondrial O_2_ consumption rate (OCR), as compared to control cells (Fig. 3s and Supplementary Fig. 11, respectively).

### Analysis of HFD adipo-barr2-KO mice kept at thermoneutrality

**Fig. 4:**
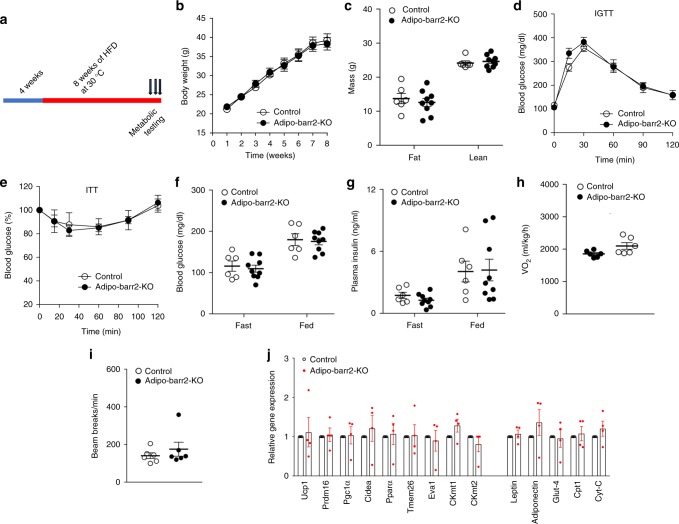
HFD Adipo-barr2-KO mice show no metabolic changes at thermoneutrality. **a** Experimental time line. When control and adipo-barr2-KO mice reached 4 weeks of age, they were maintained on a HFD at thermoneutrality (30 °C). **b** Body weights of control and adipo-barr2-KO mice maintained on HFD at thermoneutrality (*n* = 6–9 per group). **c** Body composition of mice fed with HFD for 8 weeks at thermoneutrality (*n* = 6–9 per group). **d** I.p. glucose tolerance test (1 g glucose/kg; IGTT) carried out after 7–8 weeks of HFD feeding at thermoneutrality (*n* = 6–9 per group). **e** Insulin tolerance test (1 U insulin/kg i.p.; ITT) performed after 7–8 weeks of HFD feeding at thermoneutrality (*n* = 6–9 per group). **f**, **g** Freely fed and fasting blood glucose (**f**) and plasma insulin (**g**) levels measured after 7–8 weeks of HFD feeding at thermoneutrality (*n* = 6–9 per group). **h**, **i** O_2_ consumption rate (**h**) and ambulatory activity (**i**) at thermoneutrality after 7–8 weeks of HFD feeding (*n* = 6–9 per group). **j** Relative transcript levels of browning/beiging marker genes and other key metabolic genes determined by qRT-PCR analysis of iWAT RNA after 7–8 weeks of HFD feeding at thermoneutrality (*n* = 4 per group). All data are expressed as means ± s.e.m. Male mice were used for all the experiments

At typical housing conditions (22–24 °C; ‘room temperature’), mice show considerable sympathetic nervous system (SNS) activity in order to maintain their body temperature^43^. In contrast, SNS activity is relatively low at thermoneutrality (30 °C)^43^. We found that HFD adipo-barr2-KO mice and control littermates showed similar body weight gain when raised at 30 °C (Fig. 4a, b). Similarly, no significant differences in lean or fat body mass (Fig. 4c), glucose tolerance (Fig. 4d), whole body insulin sensitivity (Fig. 4e), blood glucose and plasma insulin levels (Fig. 4f, g), oxygen consumption rate (Fig. 4h), and ambulatory activity (Fig. 4i) were observed between the mutant and control mice. Moreover, the pronounced increase in the expression of marker genes indicative of iWAT browning/beiging (*Ucp1*, *Prdm16*, *CKmt2*, and etc.) observed at room temperature with HFD adipo-barr2-KO mice was absent when mice were housed at thermoneutrality (Fig. 4j). These data are in agreement with the notion that the beneficial metabolic phenotypes displayed by HFD adipo-barr2-KO mice are dependent on the activity of the SNS (enhanced β3-AR signaling in adipocytes).

### Analysis of propranolol-treated HFD adipo-barr2-KO mice

**Fig. 5:**
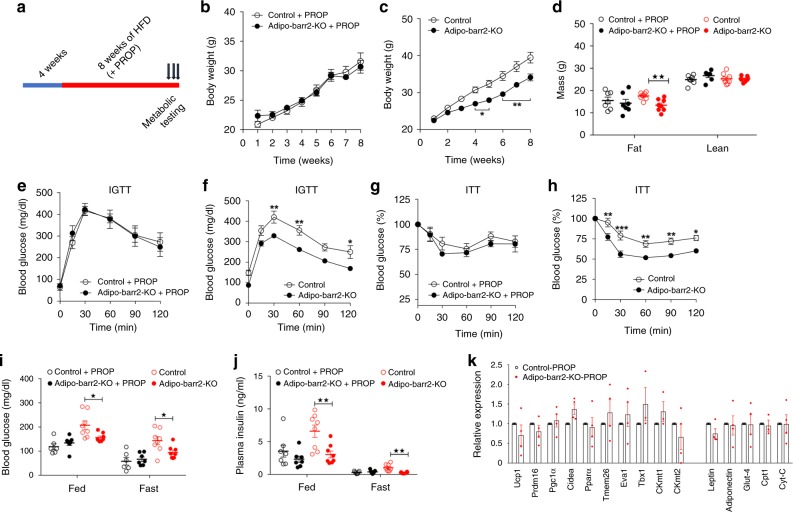
In the presence of propranolol, HFD adipo-barr2-KO mice show no metabolic phenotypes. **a** Study design. When control and adipo-barr2-KO mice were 4 weeks old, they were maintained on a HFD at room temperature (23 °C) for at least 8 weeks. During HFD feeding, a subgroup of mice received drinking water containing propranolol (PROP) at 0.5 g/l. **b**, **c** Body weights during HFD feeding. In **b**, the drinking water was supplemented with PROP. In **c**, the mice received regular drinking water for control purposes (*n* = 6–8/group). **d** Body composition of mice consuming HFD for 10 weeks with or without PROP treatment (*n* = 6–8/group). **e**, **f** I.p. glucose tolerance test (1 g glucose/kg; IGTT) in PROP-treated (**e**) or untreated (**f**) mice after 8 weeks of HFD feeding (*n* = 6–8/group). **g**, **h** I.p. insulin tolerance test (0.75 U insulin/kg i.p.; ITT) in PROP-treated (**g**) or untreated (**h**) mice after 9 weeks of HFD feeding (*n* = 6–8/group). **i**, **j** Freely fed and fasting blood glucose (**i**) and plasma insulin (**j**) levels in PROP-treated or untreated mice after 8–9 weeks of HFD feeding (*n* = 6–8/group). **k** Relative transcript levels of browning/beiging marker genes and other key metabolic genes determined by qRT-PCR analysis of iWAT RNA after 12 weeks of HFD feeding of PROP-treated mice (*n* = 4 per group). Male mice were used for all studies. Date are shown as means ± s.e.m. *p < 0.05, **p < 0.01, ***p < 0.001 (**b**, **c**, **e**–**h** two-way ANOVA followed by Bonferroni’s post hoc test.; **d**, **i**, **j** two-tailed Student’s *t*-test)

We also placed 4-week-old adipo-barr2-KO mice and control littermates on a HFD for 8 weeks in the presence of propranolol, an antagonist of all β-AR subtypes (propranolol was added to the drinking water at a concentration of 0.5 g/kg) (Fig. 5a). In parallel, another set of KO and control mice was subjected to the same experimental protocol except that the drinking water lacked propranolol. When HFD adipo-barr2-KO mice and control mice were treated with propranolol, the two groups did not show any significant differences in body weight gain (Fig. 5b), lean or fat body mass (Fig. 5d), glucose tolerance (Fig. 5e), insulin sensitivity (Fig. 5g), blood glucose or plasma insulin levels (Fig. 5i, j), and expression levels of iWAT thermogenic marker genes (Fig. 5k). As expected, when HFD adipo-barr2-KO and control mice were not exposed to propranolol, the mutant animals showed significantly reduced body weight gain (Fig. 5c), reduced fat mass (Fig. 5d), improved glucose tolerance (Fig. 5f), enhanced insulin sensitivity (Fig. 5h), and reduced blood glucose or plasma insulin levels (Fig. 5i, j). These data clearly indicated that the beneficial metabolic changes displayed by HFD adipo-barr2-KO mice at room temperature required intact β-AR signaling.

### Analysis of HFD adipo-barr2-PRDM16 double KO mice

**Fig. 6:**
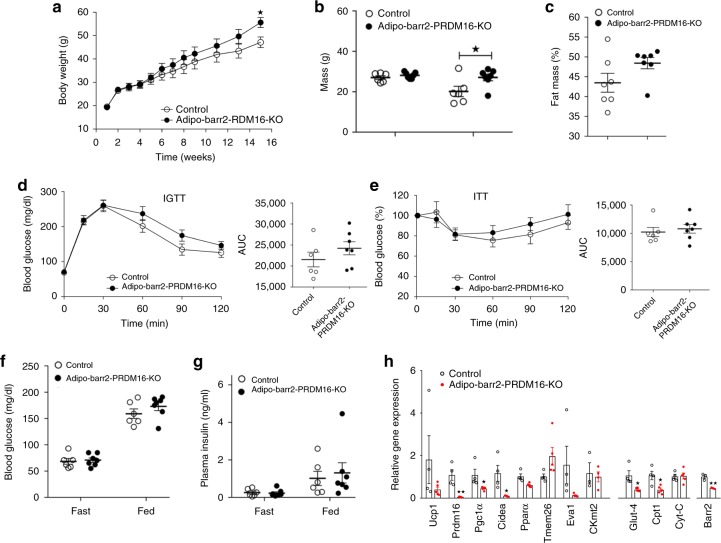
The beneficial metabolic effects displayed by adipo-barr2-KO mice are absent in barr2-PRDM16 double KO mice. **a** Body weights of control mice (genotype: *barr2 f/f Prdm16 f/f*) and adipo-barr2-PRDM16 double KO mice (genotype: *adipoq-Cre barr2 f/f Prdm16 f/f*) maintained on a HFD after 4 weeks of age (*n* = 6 or 7 per group). **b**, **c** Body composition (**b**) and fat mass (% of body weight) (**c**) of the indicated mouse strains maintained on a HFD for 15 weeks. **d**, **e** IGTT (1 g glucose/kg i.p) (**d**) and ITT (0.75 U insulin /kg i.p.) (**e**). Mice were maintained on a HFD for 8–9 weeks (*n* = 6 or 7 per group). AUC, area under the curve. **f**, **g** Freely fed and fasting blood glucose (**f**) and plasma insulin levels (**g**) in mice maintained on a HFD for 10 weeks (*n* = 6 or 7 per group). **h** Relative transcript levels of browning/beiging marker genes and other key metabolic genes, including *barr2*, determined by qRT-PCR analysis of iWAT RNA after 16 weeks of HFD feeding (*n* = 4 per group). Male mice were used for all studies. Data are expressed as means ± s.e.m. *p < 0.05, **p < 0.01 (**a** two-way ANOVA followed by Bonferroni’s post hoc test; **b**, **c**, **f**–**h** two-tailed Student’s *t*-test)

PRDM16 is a transcriptional regulatory protein that is required for the conversion of white to beige fat^7,11,14^. For example, recent studies have shown that adipocyte-specific deletion of PRDM16 prevents the browning/beiging of WAT^12^. To study whether the metabolic phenotypes displayed by HFD adipo-barr2-KO mice required the beiging of WAT, we generated adipocyte-specific barr2-PRDM16 double KO mice (*adipoq-Cre Prdm16 f/f barr2 f/f* mice). While HFD adipo-barr2 single KO mice showed a significant reduction in body weight (Fig. 1c, f), the HFD double KO mice exhibited a slight increase in body weight, as compared to the corresponding HFD control group ((*Prdm16 f/f barr2 f/f* mice) (Fig. 6a). A similar increase in body weight was observed with adipocyte-specific PRDM16 single KO mice^12^. This increase in body weight was associated with a significant increase in body fat mass (Fig. 6b; note that HFD adipo-barr2-KO mice showed a decrease in body fat mass). Moreover, the improvements in glucose tolerance and insulin sensitivity observed with HFD adipo-barr2-KO mice (Fig. 2a, b) were absent in HFD adipo-barr2-PRDM16 double KO mice (Fig. 6d, e). In addition, while HFD adipo-barr2-KO mice showed reduced blood glucose and plasma insulin levels (Fig. 2c, d), this effect was no longer observed in the HFD double KO mice (Fig. 6f, g). As expected due to the lack of PRDM16, the expression of several key thermogenic maker genes was drastically reduced in iWAT of HFD adipo-barr2-PRDM16 double KO mice (Fig. 6h).

Taken together, these data strongly suggest that the beneficial metabolic phenotypes displayed by the HFD adipo-barr2-KO mice require the beiging of WAT.

### *BARR2* expression is human subcutaneous fat

We also examined whether *BARR2* expression was altered in subcutaneous fat of obese subjects suffering from insulin resistance (for patient data, see Supplementary Table 1). Interestingly, qRT-PCR studies showed that *BARR2* transcript levels were significantly increased in subcutaneous adipose tissue isolated from obese individuals, as compared to the corresponding *BARR2* mRNA levels obtained with samples from age-, race-, and sex-matched individuals who did not suffer from obesity and insulin resistance (Supplementary Fig. 12). These data suggest the possibility that increased BARR2 activity reduces β-AR signaling in adipose tissue of obese subjects, thus contributing to the pathophysiological changes associated with increased adiposity.

## Discussion

In the present study, we demonstrated that adipocyte-specific barr2 KO mice displayed pronounced metabolic improvements when maintained on a HFD. These effects included reduced body weight gain and body fat mass, decreased blood glucose levels, and increases in glucose and insulin tolerance (Figs. 1, 2). We also showed that these metabolic improvements were associated with the beiging of white fat (Fig. 3d, e), a pronounced upregulation of many browning/beiging-associated marker genes in white fat (iWAT) (Fig. 3a–c), and an increase in energy expenditure (Fig. 1h, i). Food intake was unaltered in HFD adipo-barr2-KO mice (Fig. 1l). It is likely that the observed improvements in blood glucose homeostasis and insulin sensitivity are secondary to the decrease in adiposity displayed by the HFD adipo-barr2-KO mice. Consistent with this notion, the plasma levels of several proinflammatory adipokines were significantly reduced in HFD adipo-barr2-KO mice (Fig. 2h–k).

Recent work has shown that adipocyte-specific deletion of PRDM16 prevents the browning/beiging of WAT^12^. To test the hypothesis that the beneficial metabolic phenotypes displayed by HFD adipo-barr2-KO mice required the beiging of WAT, we generated and analyzed adipo-barr2-PRDM16 double KO mice. We found that essentially all beneficial metabolic effects caused by adipocyte barr2 deficiency, including decreased body fat mass and increased glucose and insulin tolerance, were no longer observed with HFD adipo-barr2-PRDM16 double KO mice (Fig. 6). This observation strongly supports the concept that the metabolic improvements exhibited by HFD adipo-barr2-KO mice require the browning/beiging of WAT.

Activation of the SNS (e.g., by cold exposure) efficiently promotes the browning/beiging of WAT^11,13,14^. To test the potential involvement of the SNS in the metabolic changes characteristic for HFD adipo-barr2-KO mice, we housed the mutant mice, along with their control littermates, at thermoneutrality (30 °C). At thermoneutrality, the activity of the SNS is significantly reduced, as compared to room temperature^43^. Strikingly, the beneficial metabolic phenotypes displayed by HFD adipo-barr2 KO mice were no longer detectable at thermoneutrality (Fig. 4), providing additional evidence that the improved metabolic status caused by the lack of barr2 in adipocytes is dependent on the activity of the SNS.

Activation of the SNS leads to the release of NE from sympathetic nerve endings which affects adipocyte function by activating β-ARs^30^. To demonstrate the potential involvement of β-ARs in the observed metabolic changes, we treated HFD adipo-barr2 KO mice and their control littermates with propranolol, an antagonist of all three β-ARs. Strikingly, propranolol treatment abolished all beneficial metabolic effects caused by the barr2 mutation (Fig. 5), clearly indicating that β-AR activity is essential for the phenotypic changes displayed by HFD adipo-barr2 KO mice.

All three β-ARs are expressed by mouse adipocytes, although the β3-AR represents the predominant β-AR receptor subtype (β3 > β2 > β1)^30^. Functional assays with subtype-selective β-AR agonists demonstrated that barr2 deficiency selectively enhanced signaling through β3-ΑR (Fig. 3l–n).

Recent work by the Spiegelman group identified a UCP1-independent thermogenic pathway that involves the stimulation of adipocyte creatine metabolism, promoting ATP production and consumption in mitochondria from beige fat^16,17^. It is likely that this pathway plays a key role in mediating the beneficial metabolic phenotypes exhibited by HFD adipo-barr2 KO mice. First, *CKmt2* RNA and CKmt2 protein levels were dramatically increased in iWAT from HFD adipo-barr2 KO mice (CKmt2 is a key enzyme of a futile creatine cycle that is operative in beige fat)^17^ (Fig. 3c, d). Second, CK activity was greatly increased in mitochondria prepared from barr2-deficient iWAT, as compared to control preparations (Supplementary Fig. 7).

As discussed above, we convincingly demonstrated that barr2 acts as a potent inhibitor of β3-AR signaling in adipocytes. Given previous findings that β3-AR agonists can prevent or even reverse obesity and diabetes in various animal models^13,30,44^, our new observations are of particular interest.

Studies with various cell lines demonstrated that the β3-AR, in contrast to the other two β-AR subtypes and most other GPCRs, is resistant to short-term, agonist-promoted internalization^31,33,34^, most likely due to the lack of intracellular phosphorylation sites required for the recruitment of β-arrestins^34^. Consistent with this observation, agonist-activated β3-ARs failed to interact with β-arrestins, as studied via BRET or other experimental strategies^31,32^.

In the present study, we report the unexpected finding that selective stimulation of the β3-AR expressed by cultured mouse adipocytes led to a rapid and robust internalization of β3-ARs (Fig. 3p, q). Moreover, agonist-induced β3-AR internalization was abolished following siRNA-mediated knockdown of *barr2* in these cells (Fig. 3p), indicating that β3-AR internalization was mediated by barr2. These observations clearly demonstrate that β3-AR internalization is cell-type-specific. We speculate that barr2-mediated β3-AR internalization requires additional cofactors that are only expressed in certain cell types such as adipocytes. Clearly, this topic remains to be studied in greater detail in future studies.

It should be mentioned in this context that several mutant GPCRs engineered to lack intracellular phosphorylation sites retained the ability to interact with β-arrestins in an agonist-dependent fashion. Such GPCRs include, for example, the protease-activated receptor-1^45^, the leutropin receptor^46^, and the leukotriene B4 receptor 1^47^. These observations further support the concept that agonist-induced GPCR phosphorylation is not an absolute requirement for β-arrestin recruitment.

Many studies have shown that structurally different agonists can stabilize different GPCR conformations, which differ in their ability to activate G proteins and/or to bind β-arrestins^48–50^. This finding has greatly stimulated the search for novel, so-called ‘biased’ agonists that lead to the preferential activation of G proteins or the selective recruitment of β-arrestins, respectively. As discussed in detail elsewhere, certain functionally biased agonists may become clinically useful drugs endowed with increased therapeutic efficacy and an improved side effect profile^49–52^. For example, several studies suggest that G protein-biased μ-opioid receptor agonists may be able to relieve pain without causing the characteristic side effects (tolerance, addiction, etc.) associated with the use of morphine and other non-biased μ-receptor agonists (for a recent review, see ref. ^52^). In a similar fashion, the development of G protein-biased β3-AR agonists that do not trigger barr2-mediated β3-AR internalization may lead to highly efficacious, novel therapeutic agents for the treatment of obesity and type 2 diabetes.

Whereas the β3-AR is the predominant β-AR receptor subtype in rodent adipocytes, human fat cells preferentially express β1- and β2-ARs^53^. However, β3-ARs are also expressed at significant (though lower) levels in human adipocytes^53,54^. In 2012, a selective β3-AR agonist, mirabegron, has been approved for the treatment of overactive bladder, due to the presence of β3-ARs on bladder smooth muscle cells. A recent proof-of-concept study demonstrated that acute treatment of healthy male individuals with a relatively high dose of mirabegron increased resting metabolic rate, and that this beneficial metabolic effect was associated with BAT activation^55^. However, earlier work had demonstrated that chronic treatment of human subjects with selective β3-AR agonists did not lead to a sustained increase in energy expenditure or body weight reduction^56,57^, perhaps due to barr2-mediated loss of cell surface β3-ARs. For this reason, the development of novel G protein-biased β3-AR agonists that do not promote barr2 recruitment may provide important new tools to chronically stimulate energy expenditure for therapeutic purposes (see previous paragraph).

In conclusion, we showed, for the first time, that barr2 is a critical regulator of adipocyte function and whole body glucose and energy homeostasis. Our findings suggest that G protein-biased β3-AR agonists may become clinically useful as novel therapeutic agents for the treatment of obesity and related metabolic disorders.

## Methods

### Drugs, reagents, commercial kits, and antibodies

All drugs, reagents, commercial kits, and antibodies and their sources are listed in Supplementary Table 2.

### Animals

Mice lacking β-arrestin-2 (*barr2;* official gene name: *arrb2*) selectively in adipocytes were generated as described in the following. Floxed *barr2* mice (*barr2 f/f* mice) were obtained on a pure C57BL/6 J background^26^. *Adipoq-Cre* mice were purchased from the Jackson Laboratories (Stock No. 010803; genetic background: C57BL/6 J). *Adipoq-Cre* mice were crossed with *barr2 f/f* mice to generate *barr2 f/f* mice hemizygous for the *adipoq-Cre* transgene and *barr2 f/f* control mice. All mice were maintained on a pure C57BL/6 J background.

Adipocyte-specific *Prdm16-barr2* double KO mice were generated by crossing *adipoq-Cre barr2 f/f* mice with floxed *Prdm16* mice (*Prdm16 f/f* mice; these mice were kindly provided by Drs. Paul Cohen and Bruce Spiegelman^12^).

All animal studies were carried out according to the US National Institutes of Health Guidelines for Animal Research and were approved by the NIDDK Institutional Animal Care and Use Committee. We complied with all relevant ethical regulations for animal testing and research.

### Mouse maintenance and diet

All experiments were carried out with male littermates. Unless stated otherwise, mice were maintained at room temperature (23 °C) on a standard chow (7022 NIH-07 diet, 15% kcal fat, energy density 3.1 kcal/g, Envigo Inc.). The mice had free access to water and food, and were kept on a 12-h light, 12-h dark cycle. In a subset of experiments, 4–5-week-old male mice were switched to a high fat diet (HFD; F3282, 50% kcal fat, energy density 5.5 kcal/gm, Bioserv). Mice consumed the HFD for at least 8 weeks, unless stated otherwise.

### Gene expression analysis using qRT-PCR

Mouse tissues were collected and frozen quickly on dry ice. Total RNA was extracted and purified using the RNeasy mini kit (Qiagen). cDNA was synthesized using SuperScript™ III First-Strand Synthesis SuperMix (Invitrogen), and quantitative PCR was performed using the SYBR green method (Applied Biosystems). RNA expression data were normalized relative to the expression of *β-actin* (*Atcb*) or 18 S rRNA using the ΔΔCt method. A complete list of primer sequences is provided in Supplementary Table 3.

### Western blotting

Frozen tissues were homogenized in adipocyte lysis buffer (50 mM Tris, pH 7.4, 500 mM NaCl, 1% NP40, 20% glycerol, 5 mM EDTA, and 1 mM phenylmethylsulphonyl fluoride (PMSF)), supplemented with complete EDTA-free protease inhibitor cocktail (Roche). Cell lysates were centrifuged twice at 12,000 × *g* for 15 min^17^. Subsequently, the supernatants were aliquoted and stored at −80 °C for further analysis. Protein concentrations were determined by using a bicinchoninic acid assay kit (Pierce). Protein lysates were denatured at 95 °C using NuPAGE LDS sample buffer (Thermo Fisher Scientific), and proteins were separated via SDS-PAGE followed by transfer to nitrocellulose membranes. Membranes were incubated overnight at 4 °C with primary antibody, followed by treatment with an HRP-conjugated anti-rabbit or anti-goat secondary antibody. Specific protein bands were visualized by using SuperSignal West Pico Chemiluminescent Substrate (Pierce). Uncropped and unprocessed scans of the most important blots are shown in Supplementary Figs. 13 and 14.

### In vivo metabolic tests

In vivo metabolic tests were performed with adult male mice that were at least 8 weeks old. Glucose tolerance tests (IGTT) were carried out using mice that had been fasted overnight for 12 h. Mice received 1 or 2 g/kg (as indicated) of glucose i.p., and blood was collected from the tail vein at specific time points (0, 15, 30, 50, 90, and 120 min post-injection). Blood glucose concentrations were determined using a portable glucometer (Contour Glucometer; Bayer). For insulin tolerance tests, mice were fasted for 5–6 h and then injected i.p. with human insulin (0.75 or 1 U/kg; Humulin, Eli Lilly). Subsequently, blood glucose levels were determined at time points similar to those used for the IGTT assay.

### Determination of plasma insulin and leptin levels

Blood was collected from the tail vein of fasted mice (12 h overnight fast) or from mice that had free access to food using blood collection tubes. Plasma was obtained by centrifuging blood samples at 4 °C for 10 min at ~12,000 × *g*. Plasma insulin concentrations were measured using an ELISA kit (Crystal Chem Inc.) by following the manufacturer’s instructions. Plasma leptin levels were measured using an ELISA kit (R&D Systems).

### Measurement of plasma adipokine and cytokine levels

Blood was collected from the mandibular/jugular vein with K_2_-EDTA-containing tubes (Microvette, Sarstedt) and then quickly centrifuged at 4 °C to collect plasma (see previous paragraph). Plasma adipokine and cytokine levels were measured using the Bio-Plex Multiplex Immunoassay System (Bio-Rad), following the manufacturer’s instructions. The Luminex Milliplex Analyzer (Luminex) was used to determine adipokine/cytokine concentrations, as described by the manufacturer.

### Differentiation of 3T3-F442A cells

3T3-F442A preadipocytes (Kerafast) were maintained in DMEM supplemented with 10% bovine calf serum (BCS) in a 10% CO_2_ incubator at 37 °C. Two days after the cells had reached confluence, they were differentiated via incubation in DMEM containing 10% BCS, insulin (0.5 μg/ml), IBMX (250 μM), TZD (2 μM rosiglitazone), dexamethasone (0.5 μM), and indomethacin (60 μM) for 48 h. Subsequently, the cells were incubated with DMEM plus 10% BCS and insulin (0.5 μM) for two days and then maintained in DMEM containing 10% BCS.

### Treatment of 3T3-F442A cells with barr2 siRNA

*Barr2* siRNA pool (SMARTpool) and scrambled control siRNA were purchased from GE Dharmacon. On day 0, 3T3-F442A cells were transfected with siRNA using Lipofectamine RNAiMAX (Thermo Fisher Scientific) by following the manufacturer’s instructions. Four hour later, cells were changed to differentiation media (see previous paragraph). On day 5, differentiated 3T3-F442A adipocytes were used for further experiments.

### Generation of differentiated white adipocytes

Subcutaneous inguinal fat (iWAT) from 8–12-week-old mice (males) was digested at 37 °C for 30–45 min in Krebs-Ringer-Hepes-bicarbonate buffer (KRH buffer) containing collagenase 1 (Sigma–Aldrich). The digested tissue suspension was filtered through a 100 μm cell strainer, followed by centrifugation at 400 × *g* for 5 min at room temperature. The cell pellet was resuspended in Dulbecco’s PBS (dPBS) and filtered through a 40 μm cell strainer. The cell suspension was centrifuged again as above to obtain a pellet containing the stromal vascular fraction (SVF). The pellet was resuspended in DMEM containing 10% FBS, supplemented with pen-strep and glutamine (200 μM). Cells were initially plated in 6-well plates. After having reached 50% confluency, cells were re-plated in Seashore XF 96 well plates at a density of 6000 cells/well. Once the cells had reached confluency, they were differentiated into white adipocytes using differentiation media containing insulin, IBMX, TZD, dexamethasone, and indomethacin (for details, see previous paragraph). After 48 h, media were replaced with DMEM containing 10% FBS and 0.5 μM insulin. About 24 h before experiments, media were replaced with DMEM supplemented with 10% FBS^41^.

### HEK cell culture and transfection

HEK 293 T cells (ATCC, American Type Culture Collection, CRL-3216) were transfected with β2- or β3-AR mammalian expression plasmids using Lipofectamine 2000 (Thermo Fisher Scientific), following the manufacturer’s instructions. The expression plasmid coding for the human β2-AR (vector backbone: pcDNA 3.1) was kindly provided by Dr. Vsevolod Gurevich (Vanderbilt Univesity). The expression construct encoding the mouse β3-AR (vector backbone: pCMV6) was purchased from OriGene Technologies Inc. Six hour after transfections, 1 × 10^5^ cells were plated in collagen-coated 24-well plates. Cells were allowed to grow for 48 h and then used for experiments.

### Body composition analysis

The lean/fat mass composition of mutant and control mice was measured using the 3-in-1 Echo MRI Analyzer (Echo Medical System).

### Propranolol treatment

Mice (4-week-old males) were placed on a HFD for 8 weeks in the presence of propranolol. Propranolol was added to the drinking water at a concentration of 0.5 g/kg^58,59^. During this period, body weights were measured once per week. At the end of HFD feeding period, the mice were subjected to various metabolic tests.

### Studies at thermoneutrality

Mice (4-week-old males) were kept at 30 °C and then maintained on a HFD for 8 weeks. Body weights were measured weekly, and metabolic tests were performed at the end of the HFD feeding period.

### Cold exposure of mice

Mice (4-week-old males) were maintained on a HFD for 12 weeks. The mice were then acclimatized to 4 °C over a period of one week by reducing the environmental temperature incrementally by 2 °C/day. After this acclimatization period, mice were kept at 4 °C for one week.

### Radioligand binding assay with cultured cells

Differentiated 3T3-F442A or HEK 293 T cells were incubated with DMEM-Hepes (50 mM) for 30 min at 37 °C in a 10% or 5% CO_2_ incubator, respectively. In a subset of experiments, 3T3-F442A cells were treated with barbadin (100 μM for 30 min)^37^, filipin (1 μg/ml for 60 min)^60^, or dynasore (80 μM for 60 min)^61^. To stimulate β-AR internalization, cells were incubated with 1 μM CL316243 (β3-AR agonist) or 10 μM isoproterenol (non-selective β-AR agonist) for 30 min at 37 °C in the same buffer. When 3T3-F442A cells were exposed to CL316243, β1- and β2-ARs were blocked with CGP-20712 (500 nM) and ICI-118551(100 nM), respectively, to ensure that CL316243 exclusively activated β3-ARs. After the 30 min agonist stimulation period, cells were washed with ice-cold DMEM-Hepes and then incubated with 1 nM of the radioligand [I^125^]CYP (specific activity: 3.7 MBq; PerkinElmer, MA) for 2 h at 4 °C in DMEM-Hepes (50 mM) binding buffer (incubation volume: 300 μl). Non-specific binding was determined as binding in the presence of propranolol (100 μM), a blocker of all β-ARs. Subsequently, the cells were quickly washed with PBS and lysed with 0.05 N NaOH. Radioactivity was counted using a Wizard Gamma Counter (PerkinElmer). The amount of radioactivity was normalized to the amount of protein present in the sample.

### Binding assay with iWAT plasma membrane preparations

Subcutaneous fat pads from iWAT were collected from mutant and control mice and homogenized in a buffer containing 250 mM sucrose, 10 mM Tris HCl, and 1 mM EDTA. Plasma membrane preparations were obtained as described^62^. In brief, homogenates were first centrifuged at 960 × *g* for 2 min at 4 °C. The supernatant was then centrifuged at 20,000 × *g* for 20 min at 4 °C. The resulting pellet was resuspended in homogenization buffer, layered on a sucrose gradient, and centrifuged at 160,000 × *g* for 60 min. The bottom layer containing the plasma membrane fraction was re-centrifuged at 150,000 × *g* for 60 min. Subsequently, the resulting pellet was dissolved in homogenization buffer. The resulting plasma membrane preparations were then used for radioligand binding studies.

Binding assays were performed as described^63^. In brief, binding buffer consisted of 50 mM HEPES, 4 mM MgCl_2_, and 0.04% BSA (total incubation volume: 200 μl). The binding mixture contained 5 μg of plasma membrane protein and 1 nM of [I^125^]CYP. To ensure that [I^125^]CYP selectively labeled β3-ARs, CGP-20712 (500 nM; β1-AR-selective blocker) and ICI-118551 (100 nM; β2-AR-selective blocker) were added to the binding mixture. Binding reactions were carried out at 30 °C for 3 h and then terminated by rapid filtration over GF/C fiber filters (Brandel), followed by several washes with ice-cold buffer containing 20 mM Tris-HCl, (pH 7.4) and 2 mM MgCl_2_. Non-specific binding was determined as binding in the presence of propranolol (100 μM). Radioactivity was counted using a Wizard Gamma Counter (PerkinElmer).

### Isolation of mouse mitochondria

Subcutaneous fat pads (iWAT) were collected from control and mutant mice and homogenized on ice using a Teflon glass homogenizer in TES buffer (250 mM sucrose, 50 mM Tris-HCl, pH 7.4, 1 mM EDTA), supplemented with protease inhibitors (Sigma) and 1 mM PMSF. All centrifugation steps were performed for 10 min at 4 °C. Homogenates were centrifuged at 1000 × *g*, and the infranatants were collected and centrifuged at 1000 × *g*. Pellets were resuspended in TES buffer and re-centrifuged at 1000. The supernatants were then centrifuged at 10,000 × *g* to pellet down crude mitochondria. Pellets were suspended in resuspension buffer (250 mM sucrose, 5 mM Tris-HCl, pH 7.4, 0.5 mM EDTA), supplemented with protease inhibitors (Sigma) and 1 mM PMSF, and re-centrifuged at 8500 × *g*. The final pellets were suspended in 100 μl of resuspension buffer and used for experiments.

### Measurement of mitochondrial DNA content

Total DNA was extracted from iWAT of HFD adipo-barr2-KO and control mice using a Qiagen DNA extraction kit. One ng of total DNA was used to determine the ratio of mitochondrial cyclooxygenase (*CoxII*) to nuclear intron of *β-globin* by real-time PCR^64^. Primer sequences are shown in Supplementary Table 3.

### RNA-seq studies

High throughput sequencing libraries were constructed from total RNA extracted from iWAT of adipo-barr2-KO and control mice maintained on HFD for 8 weeks (*n* = 3-6/group). RNAs with RIN > 8, as assessed by the Agilent 2100 Bioanalyzer system, were used to prepare transcriptome libraries using the NEBNext Ultra RNA library prep kit (New England Biolabs). High throughput sequencing was performed using a HiSeq 2500 instrument (Illumina) at the NIDDK Genomic Core Facility (NIH, Bethesda, MD). Raw reads were mapped to the mouse (mm9) genome. Differentially expressed genes were identified using EdgeR with a log2 (fold change) cutoff of ±0.58 using the Genomatix Genome Analyzer. Biological pathway and enrichment analysis was performed using Metacore (version 6.32, Thomson Reuters, New York). The RNA sequencing data that support the findings of this study have been submitted to the NCBI Sequence Read Archive under the accession code SRP187976.

### Indirect calorimetry and energy expenditure measurements

An Oxymax/CLAMS monitoring system (Columbus Instruments) was used to measure energy expenditure (O_2_ consumption and CO_2_ production), food intake, and locomotor activity (measurement of beam breaks) simultaneously in mice housed at 22 °C or 30 °C. Sampling was performed every 13 min, measuring from 12 chambers. Data were collected for 24 h after 2 days of adaptation to the chambers^65^. Diet-induced thermogenesis was tested at 22 °C by monitoring mice fed chow diet for 3 days, followed by a 4-day exposure to HFD. Water and food were provided *ad libitum*.

### Mitochondrial CK activity

CK activity was measured using mitochondria isolated from iWAT samples of cold-exposed mice by employing a commercially available kit, following the manufacturer’s instructions (Sigma). Values were normalized to protein concentrations.

### Isolation of mature adipocytes

Inguinal fat pads (iWAT) from both KO and control mice were collected and digested with KRH buffer (2% FFA-free BSA) containing collagenase 1 via incubation at 37 °C for 30–45 min. Once the tissue was fully digested, 10 ml of KRH buffer was added to prevent further collagenase activity. Cells were then filtered through a 250 μm cell strainer. After 10 min, the floating top layer containing mature adipocytes was collected. Mature adipocytes were washed twice with KRH buffer containing 5 mM EDTA and then used for lipolysis or cAMP assays.

### Tissue and urine norepinephrine (NE) levels

Tissue and urine NE levels were quantified using an ELISA kit (Rocky Mountain Diagnostics). Tissues (iWAT) were homogenized using assay buffer containing 0.01 N HCl, 1 mM EDTA, and 4 mM Na_2_S_2_O_5_. Tissue lysates were centrifuged at ~15,000 × *g* for 15 min at 4 °C to remove debris and fat. NE levels were measured in the supernatant by following the manufacturer’s instructions.

For urine NE measurements, mice were housed individually in metabolic cages for 24 h. Urine was collected over the 24 h period in tubes containing 6 N HCl and protected from light by wrapping tubes in aluminum foil. Urine NE levels were measured via ELISA, as described in the previous paragraph.

### Measurement of tissue noradrenergic turnover

The concentrations of the catecholamines NE and dopamine and their precursors or metabolites 3,4-dihydrophyphenylalanine (DOPA), 3,4-dihydroxyphenylglycol (DHPG), and 3,4 dihydroxyphenylacetic acid (DOPAC) were quantified in mouse iWAT by liquid chromatography with electrochemical detection. Frozen tissue samples were weighed and homogenized in 0.5 ml of 0.4 M perchloric acid containing 0.5 mM EDTA. Homogenates were centrifuged and supernatants collected and stored at −80 °C until assayed. Concentrations of catecholamines and their precursors or metabolites were determined after extraction from perchloric acid-treated tissue supernatants using alumina adsorption^66^. Since all DOPAC values were below the detection limit, they were not used for the final calculation of noradrenergic turnover.

### cAMP assay

Primary mouse mature adipocytes were suspended in KRH buffer containing 100 μM IBMX. Cells were aliquoted into 1.5 ml tubes and treated with increasing concentrations of CL316243 (β3-AR-selective agonist), fenoterol (β2-AR-selective agonist), xamoterol (β1-AR-selective agonist), or 100 μM of forskolin for 30 min at 37 °C. Cells were then lysed, and changes in intracellular cAMP levels were determined by using a FRET-based cAMP detection kit (cAMP dynamic 2 kit; Cisbio Bioassays)^67^. Tissue (iWAT) cAMP levels were measured using an ELISA kit (Cayman Chemical). Tissue samples were homogenized as per the manufacturer’s protocol. Tissue lysates were centrifuged at ~15,000 × *g* for 15 min at 4 °C, and cAMP levels were determined in the supernatant.

### Ex vivo lipolysis assay

Mature murine adipocytes were aliquoted into 1.5 ml tubes and treated with CL316243 (0.1 and 1 μM), isoproterenol (1 and 10 μM), or forskolin (100 μM) for 2 h at 37 °C in a water bath placed on a shaking platform (125 rpm). Subsequently, glycerol levels in the media were determined as a measure of lipolysis. Glycerol concentrations were measured by using a glycerol assay kit (Sigma).

### Adipocyte mitochondrial oxygen consumption rate

Adipocyte mitochondrial oxygen consumption rate (OCR) was measured using the Seahorse XF96 analyzer (Seahorse Bioscience Inc.). Preadipocytes were isolated from mouse iWAT and plated at a density of 6000 cells/well. Preadipocytes were differentiated into mature adipocytes by using the differentiation protocol described under “Differentiation of 3T3-F442A Cells”. Differentiated adipocytes were maintained in DMEM containing 10% FBS for 24 h and then stimulated with isoproterenol (10 μM) or CL316243 (1 μM) at 37 °C to measure increases in mitochondrial OCR.

### Collection of human subcutaneous adipose tissue

We complied with all relevant ethical regulations for work with human participants. Subjects were admitted to the Metabolic Clinical Research Unit in the Hatfield Clinical Research Center of the National Institutes of Health (Bethesda, MD) to participate in an NIDDK/NIAMS institutional review board-approved protocol (ClinicalTrials.gov identifier NCT00428987), after having given informed consent. Insulin-resistant, obese subjects and insulin-sensitive, lean subjects were matched for sex, age, and race (for details, see Table S1). None of the subjects was on any medications. Subcutaneous adipose tissue was obtained from the abdominal region via aspiration with an 18-gauge needle under local anesthesia. Fat samples were frozen immediately in liquid nitrogen and then stored at −80 °C until further use. The insulin sensitivity (SI) index of each subject was calculated using the MinMod Millennium (6.02) program based on results from a frequently sampled intravenous glucose tolerance test (FSIVGTT) performed after a 12-h overnight fast^68^. Body fat percentage was measured by dual-energy X-ray absorptiometry (Lunar iDXA, GE Healthcare, Madison, WI). RNA was isolated from human fat samples, and qRT-PCR studies were performed as described above for mouse iWAT gene expression.

### H&E staining experiments

BAT, iWAT, and eWAT were dissected from KO and control mice and fixed in formalin. H&E staining experiments were performed using standard techniques. The cell surface area of H&E-stained adipocytes was calculated using Adiposoft software from Fiji^69^.

### Statistics

Data are expressed as means ± s.e.m. for the indicated number of observations. Prior to performing specific statistical tests, we performed tests for normality and homogeneity of variance. Data were then tested for statistical significance by two-way ANOVA, followed by the indicated post hoc tests, or by using a two-tailed unpaired Student’s *t*-test, as appropriate. A p value of less than 0.05 was considered statistically significant. The specific statistical tests that were used are indicated in the figure legends.

### Study approval

All animal studies were approved by the NIDDK Institutional Animal Care and Use Committee (NIH, Bethesda, MD).

### Reporting summary

Further information on research design is available in the Nature Research Reporting Summary linked to this article.

## Supplementary information

Supplementary Information

Reporting Summary

## Data Availability

All data underlying this study are available from the corresponding author upon reasonable request.
