# Supplementary material for: Adipocyte β-arrestin-2 is essential for maintaining whole body glucose and energy homeostasis
Source: Nat Commun. 2019 Jul 3;10:2936. doi: 10.1038/s41467-019-11003-4 (PMC6610117; doi:10.1038/s41467-019-11003-4)
Supplement: Supplementary file 1 — Supplementary Information [file 41467_2019_11003_MOESM1_ESM.pdf]

Supplementary Information

Adipocyte β-arrestin-2 is essential for maintaining whole body glucose and energy homeostasis

Pydi et al.

Table of contents

Supplementary Figures 1 – 14

Supplementary Tables 1 – 3

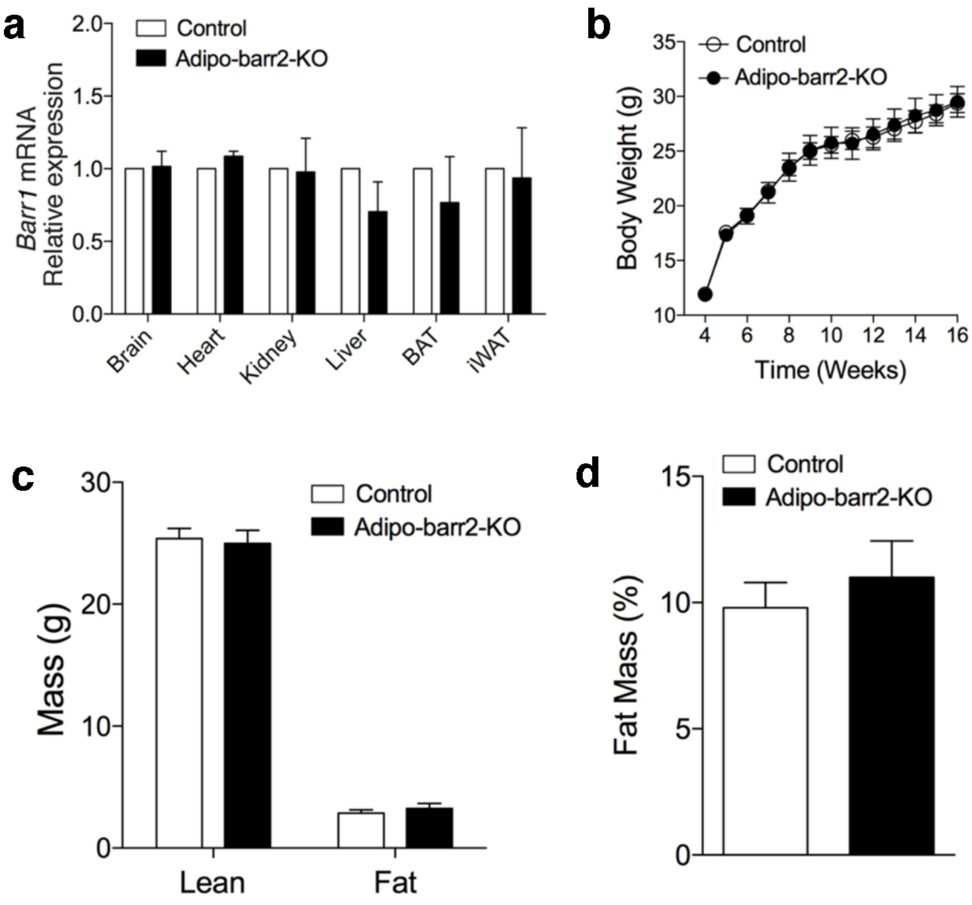

Supplementary Figure 1. Adipo-barr2-KO mice consuming regular chow do not show any changes in *barr1* expression, body weight, and body composition. (**a**) Expression of *barr1* mRNA in different tissues of adipo-barr2-KO mice and control mice maintained on regular chow (mouse age: 8-9 weeks; n=3 per group.) *Barr1* expression data are normalized relative to the expression of *β-actin*. (**b**) Body weight gain of control and adipo-barr2-KO mice consuming regular chow **(**n=7-9 per group). (**c, d**) Body composition (c) and fat mass (% of body weight) (d) of mice maintained on regular chow for 12 weeks (n=7-9 per group).

All data are expressed as means ± s.e.m. Male mice maintained on regular chow were used for all experiments.

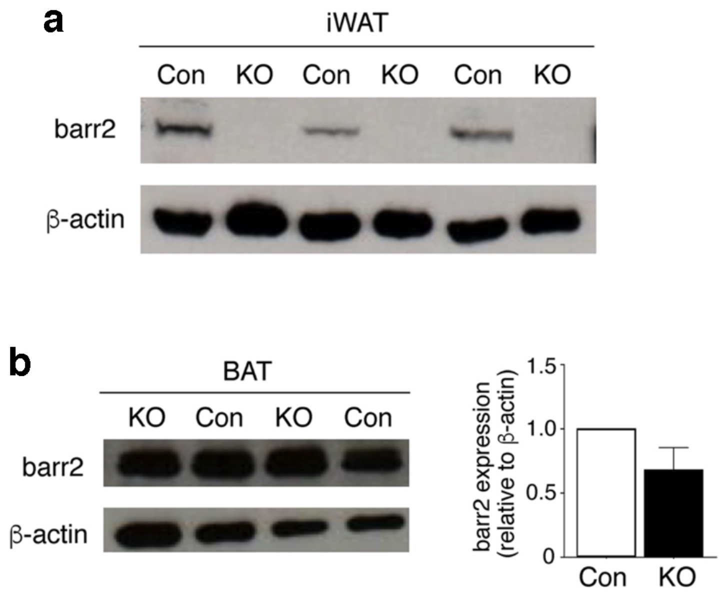

Supplementary Figure 2. Western blotting analysis of barr2 expression in iWAT and BAT of control and adipo-barr2-KO mice. (**a**) Barr2 protein is undetectable in iWAT from adipo-barr2-KO mice (KO; Con=control mice). (**b**) Barr2 protein expression levels in BAT from adipo-barr2-KO mice are not significantly reduced, as compared to control mice (n=3).

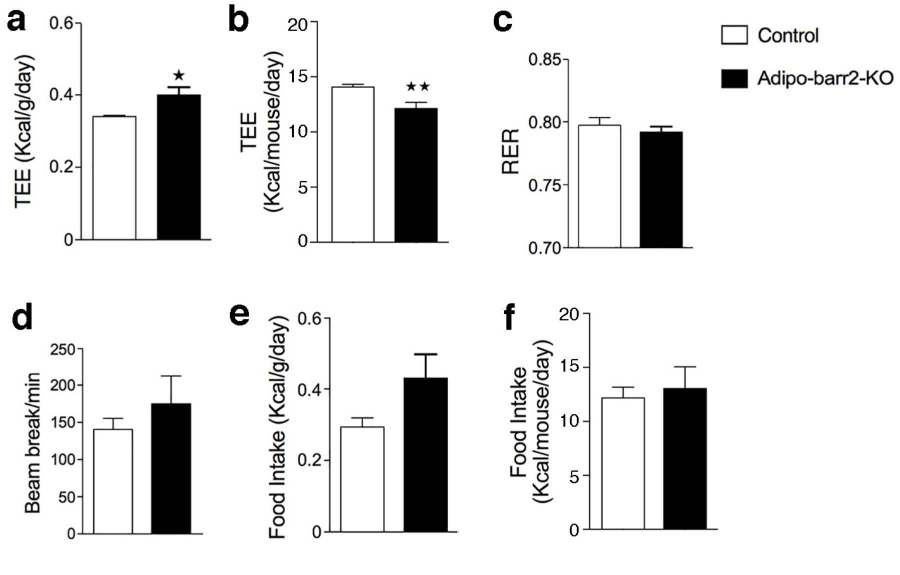

Supplementary Figure 3. Indirect calorimetry studies with control and adipo-barr2-KO mice maintained on a HFD for 8 weeks. (**a**) Total energy expenditure (TEE) normalized to body weight. (**b**) TEE per mouse. (**c**) Respiratory exchange ratio (RER). (**d**) Ambulatory activity. (**e**) Food intake normalized to body weight. (**f**) Food intake per mouse. Mice were 12 weeks old at the time of testing. Measurements were carried out using the Oxymax/CLAMS system (n=6 male mice per group). Data are expressed as means ± s.e.m. *$p<0.05$; **$p<0.01$ (two-tailed Student's t-test).

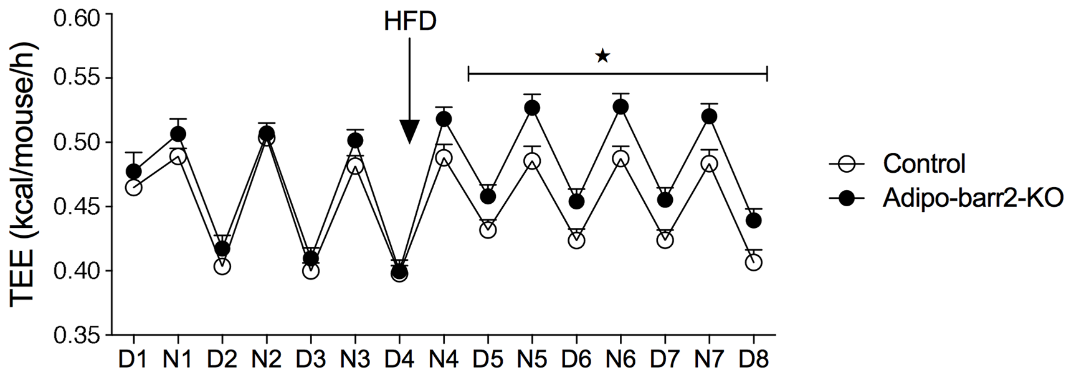

Supplementary Figure 4. Adipo-barr2-KO mice show increased total energy expenditure (TEE) after being switched to a HFD. TEE was first monitored for 3 days in 6-7-week-old mice consuming regular chow (days 1-3; D1/N1 to D3/N3), followed by TEE monitoring after switching the mice to a HFD (days 4-8; D4/N4 to D8/N8). Measurements were carried out using the Oxymax/CLAMS system. Data are expressed as means ± s.e.m (n=12 male mice per group). *p<0.05; D=day; N=night.

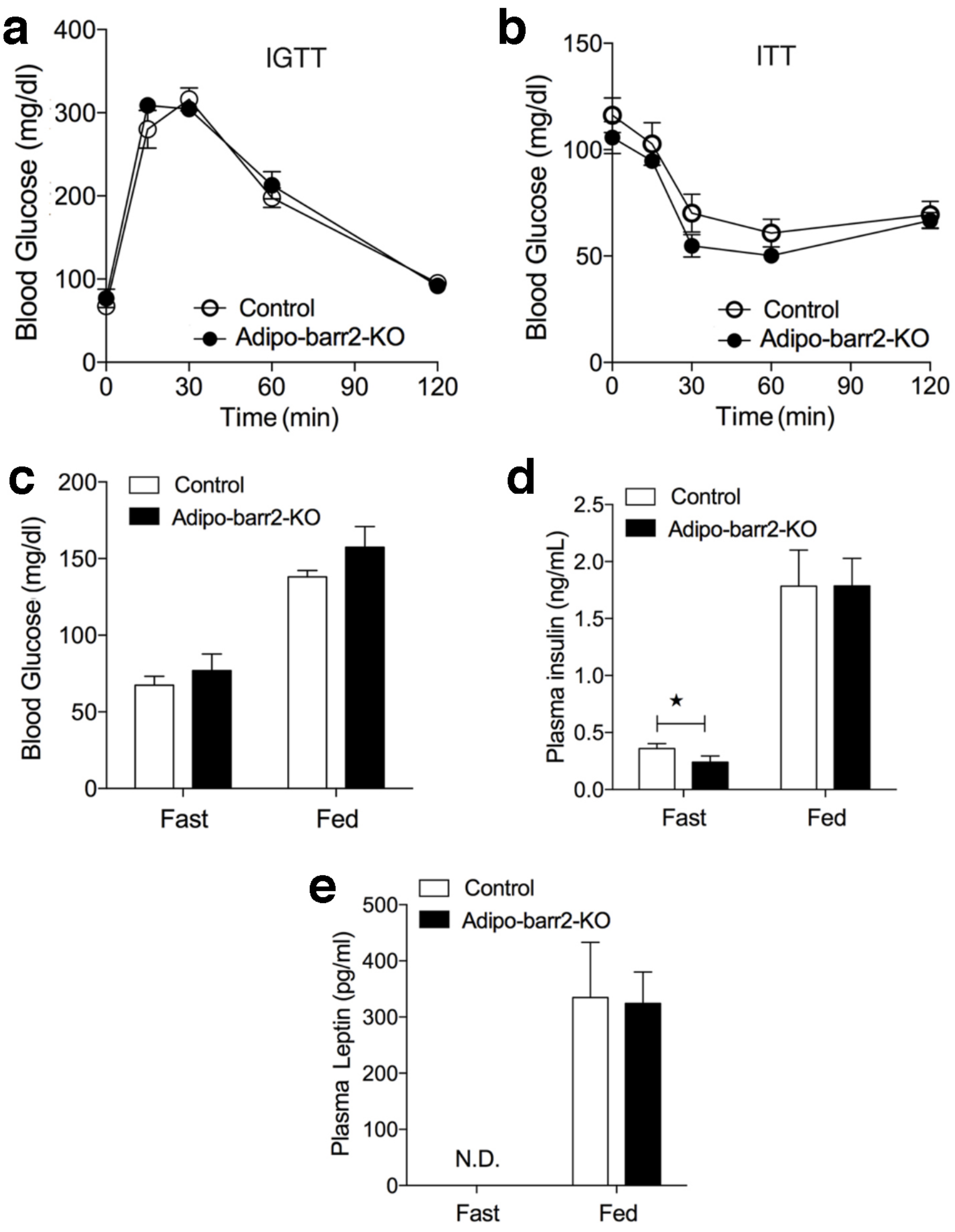

Supplementary Figure 5. Adipo-barr2-KO mice consuming regular chow do not show any major changes in glucose tolerance, insulin sensitivity, and other metabolic parameters.
(**a, b**) In vivo metabolic tests. Control and adipo-barr2-KO mice consuming regular chow were subjected to i.p. glucose tolerance (1 g glucose/kg; IGTT) (a) and insulin tolerance tests (1 U insulin/kg i.p.; ITT) (b) (n=7-9/group). (**c-e**) Fed and fasting blood glucose (c), plasma insulin (d), and plasma leptin (e) levels (n=7-10/group).
Data are given as means ± s.e.m. For all experiments, male mice were used (mouse age: 8-10 weeks). *$p<0.05$ (two-tailed Student's t-test). N.D., not determined

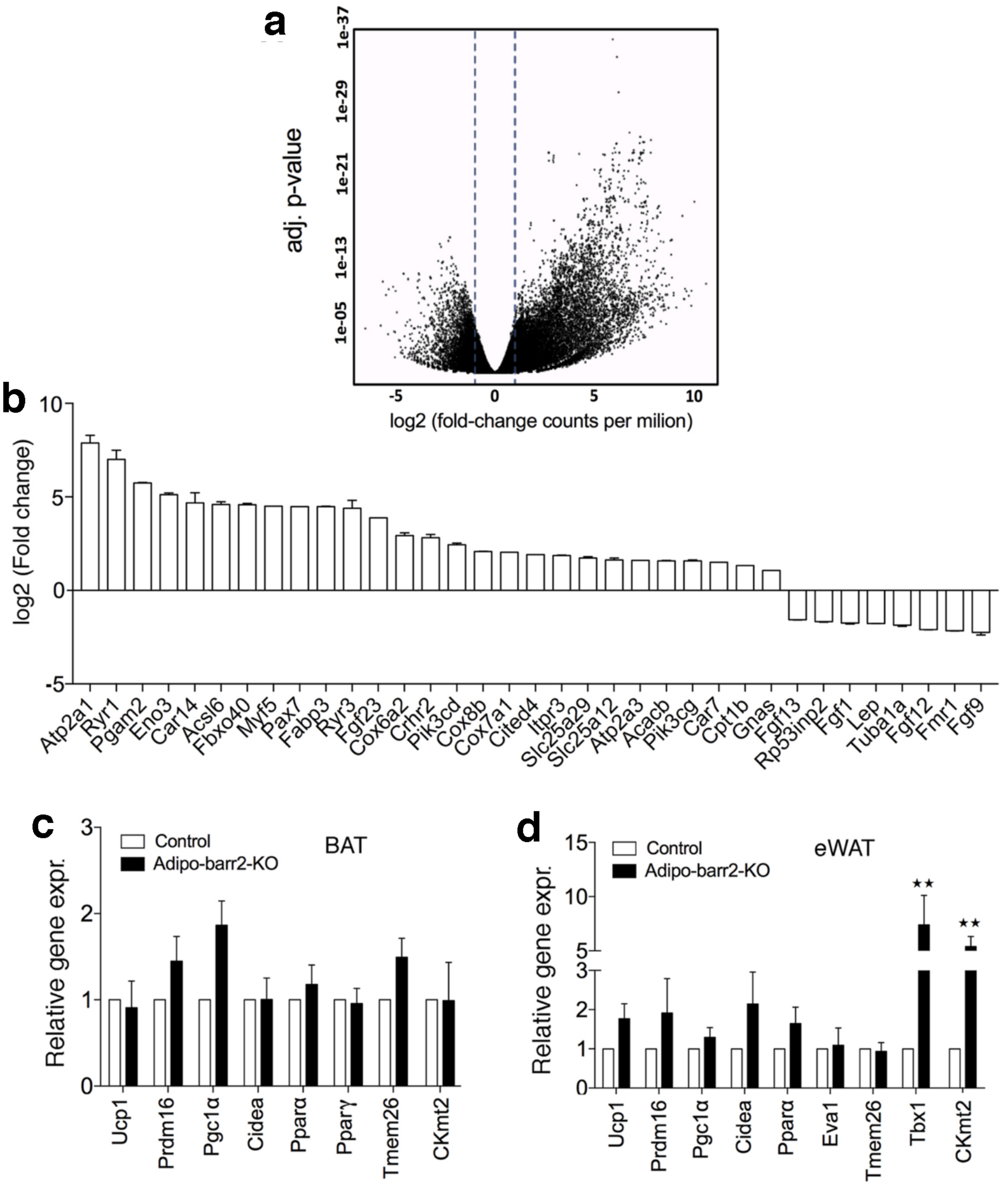

Supplementary Figure 6. Gene expression analysis using RNA from iWAT, eWAT, and BAT of HFD adipo-barr2-KO and control mice. (**a**) Volcano plot representing differentially expressed genes in iWAT of adipo-barr2-KO vs control mice, as studied via RNA-seq (n=6 per group). (**b**) Bar graph representing differences in transcript levels of key regulatory genes involved in thermogenesis, mitochondrial function, and lipid metabolism, as determined via RNA-seq. (**c, d**) Expression levels of thermogenic marker genes in BAT (c) and eWAT (d) of adipo-barr2-KO mice and control littermates, as determined by qRT-PCR. RNA was obtained from male mice that had been maintained on a HFD for 8 weeks. Data are given as means ± s.e.m. (n=4 per group). **p<0.01 (two-tailed Student's t-test).

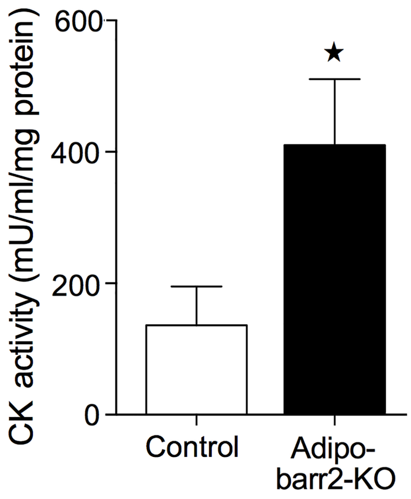

Supplementary Figure 7. Creatine kinase activity is enhanced in mitochondria from iWAT of adipo-barr2-KO mice. Male HFD adipo-barr2-KO mice and their control littermates were exposed to 4 °C for one week (n=3 per group). CK assays were carried our using mitochondria from iWAT of adipo-barr2-KO and control mice. Data are given as means ± s.e.m. *$p<0.05$ (two-tailed Student's t-test).

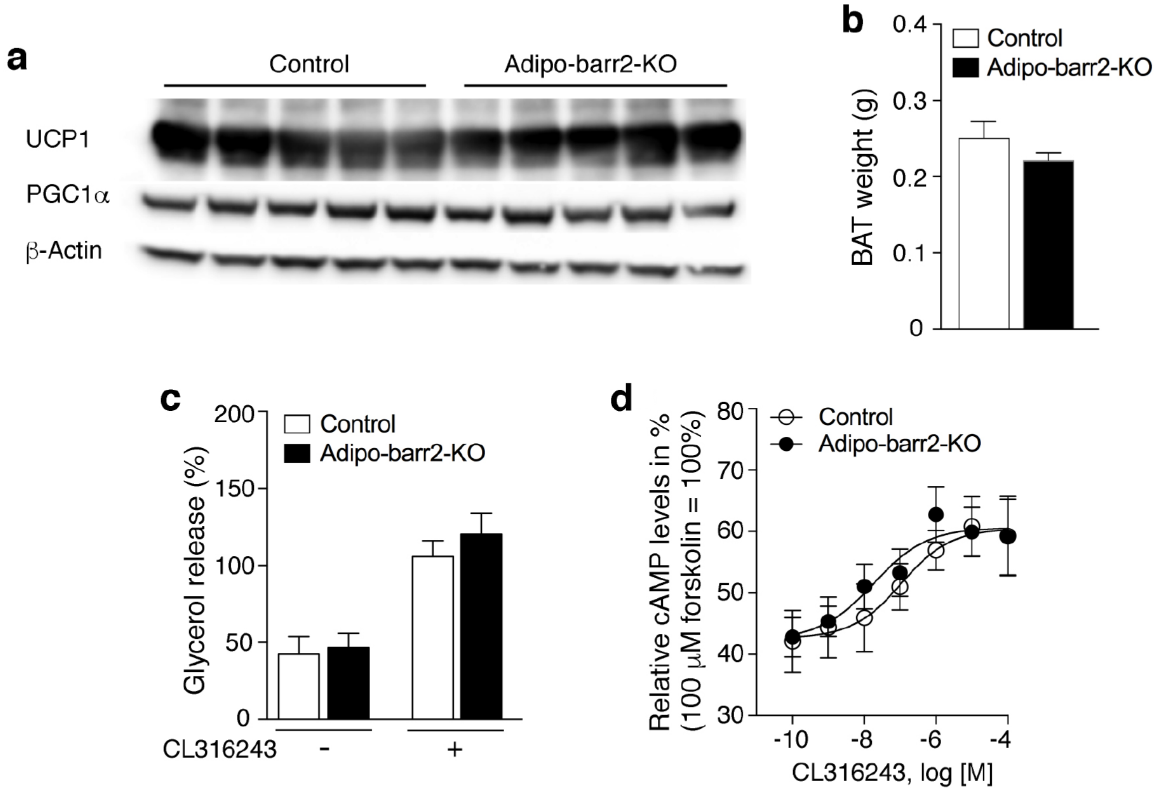

Supplementary Figure 8. Studies with BAT and BAT cells derived from adipo-barr2-KO and control mice. (**a**) Western blot analysis of UCP1 and PGC1α expression in BAT of HFD control and adipo-barr2-KO mice (n=5 per group). (**b**) BAT weight of HFD control and adipo-barr2-KO mice (n=6 per group). **(c)** CL316243 (1 μM) stimulates lipolysis in mature BAT cells prepared from mutant and control mice to a similar extent (n=3 per group). **(d)** CL316243 treatment causes comparable increases in cAMP levels in mature BAT cells prepared from mutant and control mice (n=3 per group). For all experiments, male mice were used. Both groups of mice were maintained on the HFD for 8 weeks. Data are presented as means ± s.e.m.

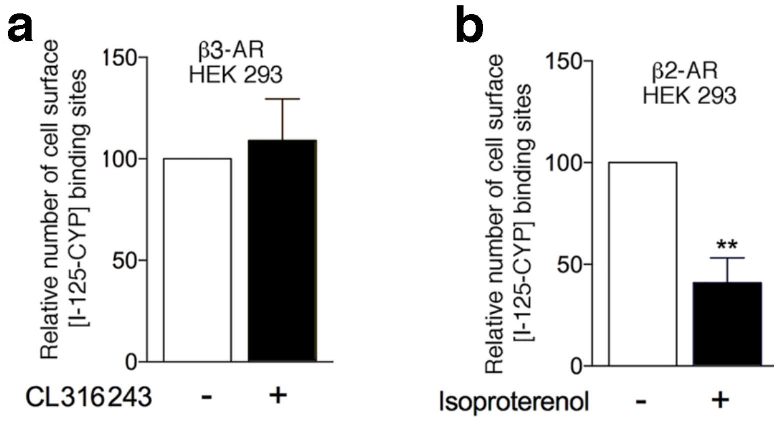

Supplementary Figure 9. β-AR internalization studies carried out with transfected HEK 293 cells. (**a**) In β3-AR-expressing HEK 293 cells, CL316243 (1 μM for 30 min) treatment does not cause β3-AR internalization. (**b**) In β2-AR-expressing HEK 293 cells, isoproterenol (10 μM for 30 min) treatment strongly promotes β2-AR internalization. Cell surface β-AR levels were determined in [I^{125}]CYP binding assays (see Methods for details). Data are given as means ± s.e.m. (n=4). **p<0.01 (two-tailed Student's t-test).

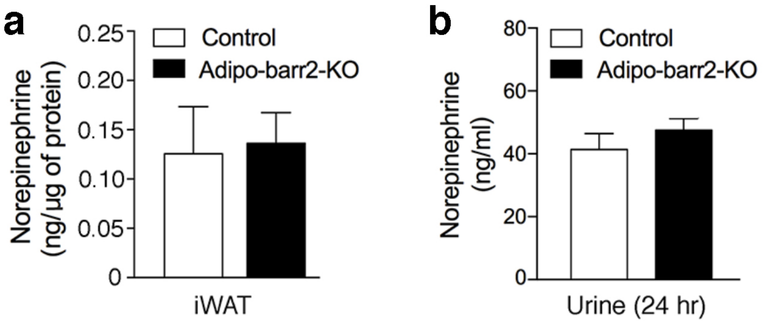

Supplementary Figure 10. HFD adipo-barr2-KO mice and control mice show similar norepinephrine levels in iWAT and urine. (**a, b**) Norepinephrine levels in iWAT (a) and in 24 hr urine (b) collected from male mice fed with a HFD for 12 weeks. Data are given as means ± s.e.m. (n=4-7 per group).

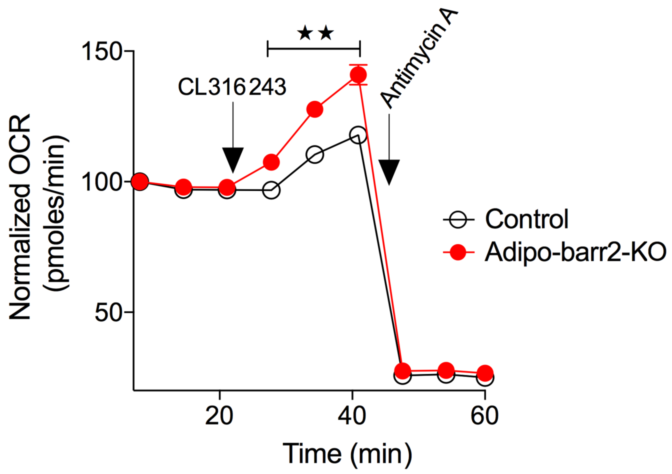

Supplementary Figure 11. Effect of CL316243 on oxygen consumption rate (OCR) of differentiated adipocytes from iWAT of control and barr2 mutant mice. CL316243 (1 μM) enhances OCR of differentiated adipocytes derived from iWAT lacking barr2, as compared to control adipocytes (n=3 per group). **p<0.01.

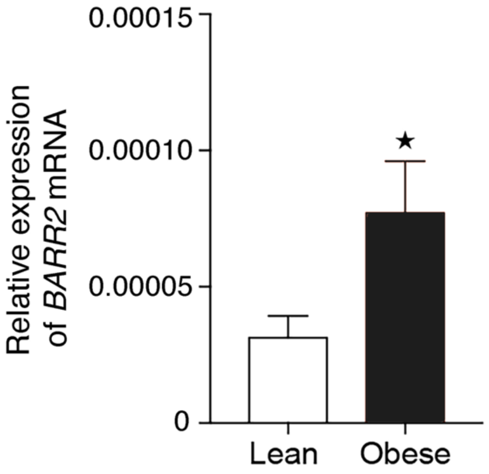

Supplementary Figure 12. *BARR2* mRNA levels are significantly increased in subcutaneous fat from obese human individuals. Relative mRNA expression of *BARR2* in subcutaneous fat from lean and obese human individuals were determined via qRT-PCR. Data were normalized relative to the expression of 18-S RNA. Data represent means ± s.e.m. (n=8 per group). *p<0.05 (two-tailed Student's t-test).

Uncropped gels for Figure 3D

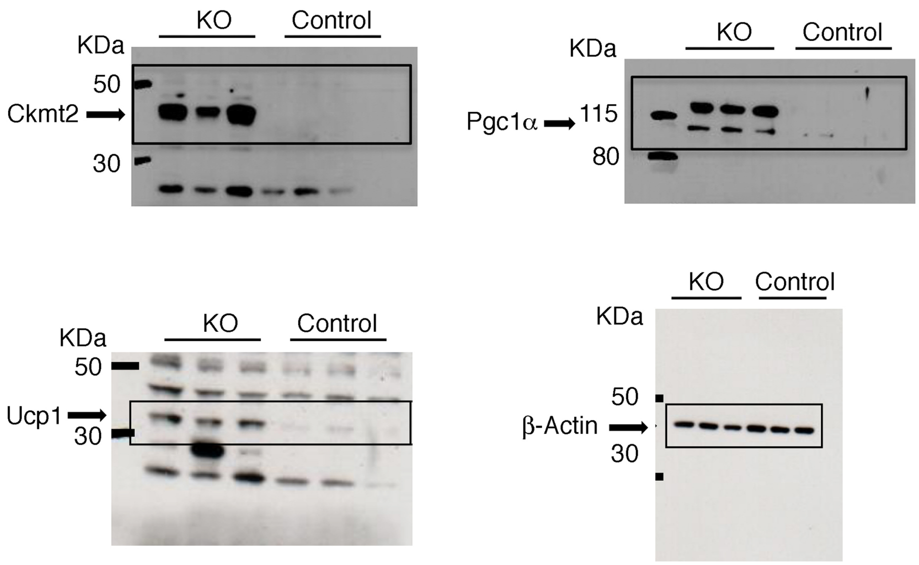

Supplementary Figure 13. Blots correspond to those shown in Figure 3d in the main manuscript.

Uncropped gels for Suppl. Fig. 2

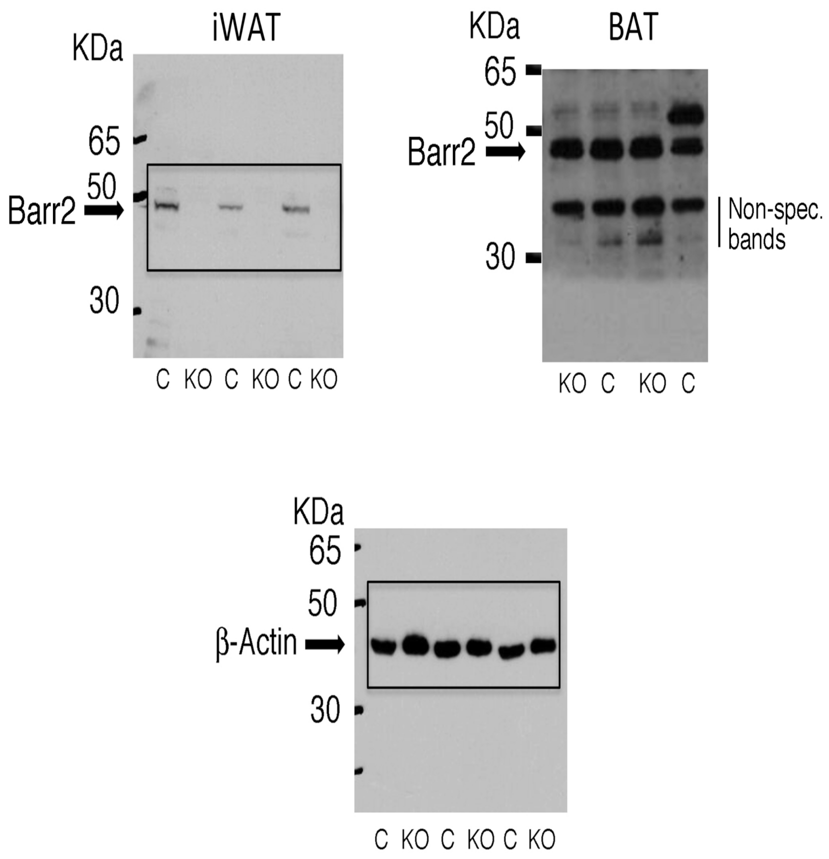

Supplementary Figure 14. Blots correspond to those shown in Supplementary Figure 2.

Supplementary Table 1 Summary of metabolic and other parameters of lean and obese subjects from which fat samples were taken for gene expression studies

Obese and non-obese subjects were matched for sex, race, and age. None of the individuals received any medications.

	**Lean**	**Obese**	**p-Value**
Sex (% female)	40%	40%	1
Race (black:white)	3:7	3:7	1
Age (years)	35.9±11.2	38.2±10.9	0.68
BMI (kg/m^2)	24.2±3.0	46.1±8.8	<0.001
Body fat (%)	23.1±9.8	46.3±6.6	<0.001
SI Index	12.2±2.3	0.90±0.55	<0.001

The SI index served as a measure of insulin sensitivity (see Methods for details).

Age, BMI, body fat (%), and SI index are given as means ± s.d. (n=8 per group). Statistical analyses were performed using two-tailed, unpaired t-tests.

Supplementary Table 2 Antibodies, drugs, reagents, and commercial kits used in the present study

	**Source**	Catalog #
**Antibodies (dilution)**		
UCP1 (1:1000)	Abcam	ab10983
PGC1α (1:1000)	Abcam	ab54481
CKmt2 (1:1000)	Abcam	ab55963
Beta-arrestin-1/2 antibody (rabbit polyclonal) (1:1000)	Provided by Dr. Vsevolod Gurevich	NA
β-Actin (1:1000)	Cell Signaling	4970S
**Drugs, reagents and commercial kits**		
CL 316,243	Sigma	Cat# C5976
BCA protein assay kit	Pierce	Cat# 23225
Iodo-(-)-Cyanopindolol, [^{125}I]	Perkin Elmer	NEX189100UC
ECL Western blotting substrate	Pierce	32106
Bovine serum albumin (fatty acid-free)	Sigma-Aldrich	Cat# A7030
Collagenase 1	Sigma-Aldrich	Cat# C0130
Triton X-100	Fisher Scientific	Cat# BP151
TWEEN 20	Fisher Scientific	Cat# BP337
QIAzol	Qiagen	Cat# 79306
Oligomycin	Sigma-Aldrich	Cat# 75351
Antimycin A	Sigma-Aldrich	Cat# A8674
FCCP	Sigma-Aldrich	Cat# C2920
Isoproterenol hydrochloride	Sigma-Aldrich	Cat# I6504
Barbadin	Toronto Research Chemicals	Cat# B118250
Filipin	Sigma-Aldrich	Cat# F9765
Dynasore hydrate	Sigma-Aldrich	Cat# D7693
ICI 118,551 hydrochloride	Tocris	Cat# 0821
CGP 20712 dihydrochloride	Tocris	Cat# 1024
Forskolin	Sigma-Aldrich	Cat# F3917
RNase-free DNase I	Qiagen	Cat# 79254
Power SYBR Green PCR Master Mix	Applied Biosystems	Cat# 4367659
RNeasy mini kit	Qiagen	Cat# 74104
DNeasy Blood & Tissue Kits	Qiagen	Cat# 69504
SuperScript™ III First-Strand Synthesis SuperMix	Invitrogen	Cat# 18080400

Complete EDTA-free protease inhibitor cocktail	Sigma-Aldrich	Cat# 11873580001
NuPAGE LDS sample buffer	Thermo Fisher Scientific	Cat# NP0007
Barr2 siRNA pool (SMARTpool)	Dharmacon	Cat# M-041022-01-0005
Lipofectamine RNAiMAX	Thermo Fisher Scientific	Cat# 13778030
Lipofectamine 2000	Thermo Fisher Scientific	Cat# 11668027
(±)-Propranolol hydrochloride	Sigma-Aldrich	Cat# P0884
Seahorse XF^e 96 FluxPak Mini	Seahorse Bioscience	Cat# 102601-100
Ultra-Sensitive Mouse Insulin ELISA Kit	Crystal Chem	Cat# 90080
Norepinephrine ELISA Kit	*Rocky Mountain* Diagnostics	Cat# BA E-5200
cAMP ELISA Kit	Cayman Chemicals	Cat# *581001*
Mouse/Rat Leptin Quantikine ELISA Kit	R&D Systems	Cat# MOB00
Bio-Plex Multiplex System	Bio-Rad	
Creatine Kinase Activity Assay Kit	Sigma-Aldrich	Cat# MAK116
NEBNext® Ultra™ RNA Library Prep Kit for Illumina®	New England Biolabs	Cat# E7530S
cAMP Dynamic 2 Kit	Cisbio Bioassays	Cat# 62AM4PE

Supplementary Table 3 PCR primers used for PCR/qRT-PCR experiments

Mouse Primers

Gene name	Forward (5'-3')	Reverse (5'-3')
Ucp1	ACTGCCACACCTCCAGTCATT	CTTTGCCTCACTCAGG ATTGG
Pgc1α	AGCCGTGACCACTGACAAC GAG	GCTGCATGGTTCTGAGTGCTAAG
Cd137	CGTGCAGAACTCCTGTGATAAC	GTCCACCTATGCTGGAGA AGG
Cidea	TGCTCTTCTGTATCGCCCAGT	GCCGTGTTAAGGAATCTG CTG
Tmem26	ACCCTGTCATCC CACAGAG	TGTTTGGTGGAGTCCT AAGGTC
Prdm16	CAGCACGGTGAAGCCATTC	GCGTGCATCCGCTT GTG
Leptin	CAAGCAGTGCCTATCCAGA	AAGCCCAGGAATGAAGTCCA
Barr1 (arrb1)	CCAAACCTTCCATGCTCAGT	TAGCCGCACAGAGTTCCTTT
Barr2 (arrb2)	AAGTCGAGCCCTAACTGCAA	GGTGAGGGTCACGAACACTT
β-actin	GATATCGCCTGCGCTGGTCGTC	ACGCAGCTCATTGTAGAAGGTGTGG
Ckmt1	TGAGGAGACCTATGAGGTATTTGC	TCATCAAAGTAGCCAGAACGGA
Ckmt2	GCATGGTGGCTGGTGATGAG	AAACTGCCCGTGAGTAATCTTG
18S rRNA	CGGCTACCACATCCAAGGAA	GCTGGAATTACCGCGGCT
Ap2	CGCAGACGACAGGAAGGT	TTCCATCCCACTTCTGCAC
Epsti1	ACCCTGATAGCACCAAACGA	AGGTCTGCCAGTTCTTGCTC
Eva1	CCACTTCTCCTGAGTTTACAGC	GCATTTTAACCGAACATCTGTCC
Tbx1	GGCAGGCAGACGAATGTTC	TTGTCATCTACGGGCACAAAG
Zic1	AACCTCAAGATCCACAAAAGGA	CCTCGAACTCGCACTTGAA
Cpt1	TTGCCCTACAGCTCTGGCATTTCC	GCACCCAGATGATTGGGATACTGT
Mcad	ATGACGGAGCAGCCAATGAT	TCGTCACCCTTCTTCTCTGCTT
Coxβ4	CTGCCCGGAGTCTGGTAATG	CAGTCAACGTAGGGGGTCATC
Cyt c	AAATCTCCACGGTCTGTTCGG	GGGTATCCTCTCCCCAGGTG
Acly	ACCCTTTCACTGGGGATCACA	GACAGGGATCAGGATTTCCTTG
Fas	GGAGGTGGTGATAGCCGGTAT	TGGGTAATCCATAGAGCCCAG
Gpt1	AGCAAGTCCTGCGCTATCAT	CTCGTGTGGGTGATTGTGAC
Acc	CATCACCATCAGCCTGGTTACA	ACTGTGTACGCTCTTCGGCAT
Srebp1	AGTGGCAAAGGAGGCACTAC	CACCCTCTGGAAGACCACA
Adiponectin	GCACTGGCAAGTTCTACTGCAA	GTAGGTGAAGAGAACGGCCTTGT
Glut4	GTGACTGGAACACTG GTCCTA	CCAGCCAGTTGCATTGTAG
Resistin	CTGTCCAGTCTATCCTTGCACAC	CAGAAGGCACAGCAGTCTTGA
Ppar-γ	GTGCCAGTTTCGATCCGTAGA	GGCCAGCATCGTGTAGATGA
Ppar-α	GCGTACGGCAATGGCTTTAT	GAACGGCTTCCTCAGGTTCTT
Barr2 f/f	ACTGACTTCCTTCCCTCCTTAA GCCCCTTGCC	CAAGCCACCAGAGTTCTCAAAGGT GGGTGTCC

Adipoq-Cre	TGGTGCATCTGAAGACACTAC	TGCTGTTGGATGGTCTTCACAG
CoxII mtDNA	GCCGACTAAATCAAGCAACA	CAATGGGCATAAAGCTATGG
β-Globin gDNA	GAAGCGATTCTAGGGAGCAG	GGAGCAGCGATTCTGAGTAGA
Prdm16 f/f	GAGCTAGGCAAGGACACTGCT	CCAGTATCAGAGAGGCAAGAA

Human primers

Gene name	Forward (5’-3’)	Reverse (5’-3’)
18S rRNA	GCAATTATTCCCCATGAACG	GGCCTCACTAAACCATCCAA
BARR1 *(ARRB1)*	TCAAGCACGAAGACACGAAC	ATGCAAGATCTCCCAACAGG
BARR2 *(ARRB2)*	CACGTCACCAACAACTCCAC	TTGTTCGAGTTGAGCCACAG
